# The impact of virtual reality exercise on motor function, balance and gait function, and cognitive function in stroke patients: an umbrella review of systematic reviews and meta-analyses

**DOI:** 10.3389/fspor.2026.1860232

**Published:** 2026-09-17

**Authors:** Xinxing Du, Yanan Guo, Yuting Chen, Jiangang Chen

**Affiliations:** 1 School of Sport and Health Sciences, Xi’an Physical Education University, Xi’an, Shaanxi, China; 2 College of Physical Education and Sport, Beijing Normal University, Beijing, China

**Keywords:** balance and gait function, cognitive function, meta-analysis, motor function, stroke rehabilitation, umbrella review, virtual reality

## Abstract

**Background:**

Stroke is a leading cause of adult disability, and most survivors experience motor, balance and gait, and cognitive impairment. Virtual reality exercise is an innovative rehabilitation approach, but fragmented evidence limits its clinical application. This umbrella review synthesized meta-analytic evidence to evaluate VR exercise effects on motor, balance/gait, and cognitive function in stroke patients.

**Methods:**

This PROSPERO-registered review CRD420261341936 followed PRISMA 2020 guidelines. Systematic reviews and meta-analyses of randomized controlled trials evaluating VR exercise in stroke rehabilitation were identified from six electronic databases. Methodological quality was assessed with AMSTAR 2, and evidence certainty classified via metaumbrella GRADE-based criteria, with quantitative evidence re-analyzed using deduplicated primary RCTs.

**Results:**

Ten meta-analyses with 367 pre-deduplication RCT entries and 14,352 total participants were included. After deduplication and exclusion of trials with unavailable or incomplete numerical data, 138, 81, and 23 unique RCTs contributed to the motor, balance/gait, and cognitive analyses, respectively. VR exercise was associated with statistically significant improvements in several functional outcomes; however, the magnitude and certainty of effects varied across domains. VR was associated with significant improvements in functional ambulation (equivalent standardized mean difference, eSMD = 0.70, 95% CI 0.23–1.17) and upper limb motor function (eSMD = 0.38, 95% CI 0.21–0.55); gait velocity (eSMD = 0.63, 95% CI 0.43–0.82) and Berg Balance Scale scores (eSMD = 0.69, 95% CI 0.45–0.92); and global cognition measured by Montreal Cognitive Assessment (eSMD = 0.48, 95% CI 0.19–0.76). Most outcomes had very low certainty (MoCA: moderate). Heterogeneity varied considerably across outcome measures (*I*^2^ ranging from 0% to 93%), and most included systematic reviews were rated as critically low methodological quality.

**Conclusions:**

VR exercise may be associated with improvements in selected post-stroke motor, balance and gait, and cognitive outcomes. However, statistically significant effects should not be interpreted as clinically meaningful benefits without established MCID thresholds or interpretable benchmarks. Evidence is further limited by substantial heterogeneity, poor review methodology, and generally low certainty. VR may therefore be a potential adjunctive rehabilitation approach, but current evidence does not support replacing conventional rehabilitation or preferential use in specific patient subgroups.

**Systematic Review Registration:**

https://www.crd.york.ac.uk/PROSPERO/view/CRD420261341936, PROSPERO CRD420261341936.

## 1 Introduction

Stroke remains the leading cause of adult disability worldwide, characterized by high incidence and high disability rates (1, 2). Beyond direct impairments in physical movement and cognitive function, which severely reduce patients’ activities of daily living and health-related quality of life, stroke imposes an enormous economic and service burden on family caregiving and the global public health system (3). Among the numerous sequelae of stroke, motor dysfunction, balance and gait abnormalities, and cognitive impairment are three core interrelated factors that determine patients’ rehabilitation outcomes and ability to reintegrate into society (4). These domains form a mutually influential network, and coordinated recovery is the cornerstone of effective stroke rehabilitation (5).

Motor function impairment—encompassing weakness, spasticity, and loss of fine motor control—is the most common sequela of stroke, affecting approximately 80% of survivors (6). Balance and gait function, which rely on the integration of motor control, sensory feedback, and postural regulation, are closely linked to motor function: deficits in lower limb motor strength directly lead to gait instability and increased fall risk, while poor balance further limits voluntary motor activities (7). Cognitive impairment, including deficits in attention, executive function, and working memory, occurs in 65%–70% of stroke patients and interacts bidirectionally with motor and balance/gait functions: cognitive deficits may hinder the learning and execution of motor skills for balance and gait control, while impaired motor function reduces sensory input to the brain, exacerbating cognitive decline (8). Therefore, comprehensive evaluation covering these three dimensions enables a more holistic reflection of patients' overall functional recovery following stroke.

Conventional rehabilitation interventions, namely physical therapy, occupational therapy and cognitive training, remain the cornerstone of post-stroke functional recovery. Nevertheless, their optimized clinical application faces multiple challenges. Functional restoration after stroke demands high-intensity, repetitive task-oriented training, yet training volume and intensity in routine clinical settings are often constrained by limited rehabilitation resources (9). Furthermore, despite individualized rehabilitation being recommended by current clinical guidelines, significant inter-individual heterogeneity exists in patients' impairment profiles and recovery trajectories, making precise personalized regimen design difficult to implement (6). In addition, prolonged repetitive rehabilitation tends to diminish patients' therapeutic motivation and sustained participation, an issue particularly prominent in long-term rehabilitation cycles (10). With the integration of digital medicine and rehabilitation engineering, VR exercise— a novel intervention combining virtual reality technology with physical activity that captures human movement trajectories and feeds them back to virtual interactive scenarios—has emerged as a promising approach in stroke rehabilitation (11). Characterized by immersive interaction, gamified motivation, and personalized adaptation, VR exercise retains the physical training value of traditional rehabilitation while strengthening brain-body coordination through multisensory stimulation and cognitive engagement. Importantly, it can simultaneously target improvements in motor control, balance coordination, and cognitive processing, addressing the core deficits of the three interrelated functional domains in stroke patients (8, 12, 13).

In recent years, numerous RCTs, systematic reviews, and meta-analyses have explored the efficacy of VR exercise in stroke patients, providing preliminary evidence of its potential benefits in improving upper limb fine motor function, lower limb gait stability, dynamic balance, and cognitive domains such as attention and executive function (12–16). However, the rapidly accumulating evidence has not yielded consistent, comprehensive consensus regarding its overall clinical benefits. Existing systematic reviews and meta-analyses mostly focus on a single functional domain: for instance, Villarroel et al. primarily evaluated VR exercise's effects on upper-extremity motor function in stroke survivors; Qi Zhang centered on patients' cognitive function and psychological well-being; Wenxin Lu explored its impacts on balance and gait. Though these studies corroborate the potential merits of VR exercise from distinct perspectives, they lack integrated assessments covering motor function, balance-gait performance and cognitive function simultaneously, resulting in fragmented evidence that fails to fully capture the multi-dimensional characteristics of post-stroke functional recovery. Meanwhile, substantial discrepancies in evidence quality and study design across secondary analyses further complicate the interpretation of available evidence. For example, Maria Krohn et al. only enrolled patients with chronic stroke and stratified interventions by low, medium and high immersion levels of VR, concluding that VR exercise improves balance and gait, yet rated the evidence for gait outcomes as low certainty. In contrast, Ming-Yu Tian et al. recruited patients across acute, subacute and chronic stroke stages, comparing immersive vs. non-immersive VR training. While confirming improvements in static postural control and dynamic balance, they noted substantial statistical heterogeneity and high risk of bias for several outcomes, urging cautious interpretation of their findings. Notably, prominent disparities exist in the evidence evaluation frameworks adopted by different meta-analyses. Villarroel et al. employed the JBI critical appraisal checklist to assess methodological quality of primary studies and applied the GRADE framework to grade evidence certainty. Tian et al. combined the PEDro scale with the OCEBM hierarchy for study quality assessment, alongside GRADE for evidence grading. By comparison, Chenli Lin et al. solely adopted the Cochrane risk-of-bias tool to evaluate primary research, utilizing funnel plots, Egger's test, the *I*^2^ statistic and *Q*-test to assess publication bias and statistical heterogeneity respectively, without standardized grading of overall evidence certainty. Zhang et al. integrated the Cochrane risk-of-bias tool with the PEDro scale for study quality appraisal, yet also omitted subsequent GRADE evidence grading. These inconsistencies indicate a lack of unified frameworks for methodological quality evaluation, risk-of-bias assessment and evidence certainty rating across meta-analyses, hindering standardized cross-study comparisons and limiting comprehensive judgment of VR exercise's overall clinical value.

As an advanced evidence synthesis method for aggregating secondary research, umbrella reviews systematically summarize findings from relevant systematic reviews and meta-analyses within a specific field, quantitatively evaluate the strength of associations between interventions and diverse outcomes, and conduct unified grading of overall evidence certainty, thereby generating high-level evidence to guide clinical practice and future research (17). Based on this rationale, the present umbrella review was conducted with three primary objectives. First, to comprehensively summarize meta-analytic evidence regarding the effects of virtual reality-based exercise on core functional outcomes in stroke rehabilitation and systematically characterize its potential health benefits across different outcome domains. Second, to evaluate the methodological quality and certainty of evidence of the included meta-analyses using a standardized assessment framework. Third, to perform subgroup analyses according to VR modality and stroke stage to systematically explore potential sources of clinical and methodological heterogeneity. In addition, rather than relying solely on the pooled effect estimates reported in previous meta-analyses, this study further extracted quantitative data from the original randomized controlled trials included in the eligible reviews and synthesized these data using a unified statistical framework to improve the accuracy and reliability of the cumulative evidence. The findings of this study are expected to provide a more robust evidence base for the design of future high-quality clinical trials and the optimization of VR-based rehabilitation strategies.

## 2 Methods

This umbrella review (overview of systematic reviews with meta-analyses) was registered in the International Prospective Register of Systematic Reviews (PROSPERO, registration number CRD420261341936). The review was conducted and reported in strict accordance with the Preferred Reporting Items for Overviews of Reviews (PRIOR) statement (18) and the Preferred Reporting Items for Systematic Reviews and Meta-Analyses (PRISMA) 2020 guidelines (19). No ethical approval was required as this study was based on published systematic reviews. This study aimed to comprehensively search and include systematic reviews and meta-analyses based on RCTs investigating the effects of VR exercise on key health-related outcomes (motor function, balance and gait function, cognitive function) in stroke patients, systematically synthesize the magnitude and direction of effect sizes from existing meta-analytic evidence, and provide high-level evidence-based support for the standardized application of VR exercise in clinical stroke rehabilitation.

### 2.1 Inclusion criteria

The inclusion criteria were developed in strict accordance with the Population, Intervention, Comparison, Outcome, Study design (PICOS) framework. All included systematic reviews and meta-analyses must meet the following criteria:

#### 2.1.1 Study population

Included studies focused on participants aged ≥18 years with a confirmed diagnosis of stroke (verified by cranial computed tomography or magnetic resonance imaging and consistent with criteria established by the World Stroke Organization or World Health Organization). All stroke subtypes (ischemic, hemorrhagic, mixed) and all post-stroke phases:(acute ≤1 month, subacute 1–6 months, chronic > 6 months) were included. Exclusions: participants <18 years; non-stroke etiologies (traumatic brain injury, spinal cord injury, Parkinson's disease); severe comorbidities precluding VR exercise.

#### 2.1.2 Intervention

Included studies evaluated VR exercise as the core intervention. VR systems can be classified into 3 major categories. These categories—non-immersive, immersive, and semi-immersive—are classified according to the degree of user immersion and the type of interface hardware used, consistent with established VR system taxonomy (20). Eligible VR exercise was described across three independent dimensions: degree of immersion, intervention combination mode, and delivery setting.

1. Classification by degree of immersion (core technical classification, basis for prespecified subgroup analysis):

Immersive VR exercise: Administered via head-mounted displays (HMDs) or cave automatic virtual environment (CAVE) systems, with task-oriented active training targeting at least one core functional domain. Included studies must explicitly report standardized intervention parameters (single session duration, weekly frequency, intensity, total cycle).

Semi-immersive VR exercise: Hybrid active training delivered via large curved screens or projection systems, which provide a high level of environmental immersion while allowing participants to perceive parts of the real environment;

Non-immersive VR exercise: Administered via desktop platforms, screen projection, tablets, or validated commercial rehabilitation gaming systems, with active exercise participation (excluding passive observation). Included studies must clarify VR equipment and core training components.

2. Classification by intervention combination mode

VR exercise alone: The intervention group received only VR exercise rehabilitation without additional conventional rehabilitation regimens;

VR exercise combined with conventional rehabilitation: VR exercise rehabilitation was applied as an adjunct intervention on top of guideline-recommended conventional stroke rehabilitation, including physical therapy, occupational therapy, gait training, and cognitive training.

Across the included systematic reviews, VR interventions were evaluated under different comparator frameworks, including VR delivered in addition to conventional rehabilitation compared with conventional rehabilitation alone, and VR compared directly with conventional rehabilitation. As these comparison frameworks address different clinical questions and were not consistently distinguished across the available meta-analytic evidence, the present review synthesized effect estimates at the outcome level rather than stratifying quantitative analyses solely by comparator type. Variation in comparator frameworks was considered a potential source of clinical heterogeneity and was incorporated into the interpretation of the pooled estimates.

3. Classification by delivery setting

Institutional VR exercise: Training completed under the guidance of rehabilitation professionals in hospitals, rehabilitation centers, or other medical facilities;

Home/community-based VR exercise: Training completed independently by patients or with assistance from caregivers in home or community settings.

#### 2.1.3 Control

Eligible studies were required to adopt a parallel control design. The following three types of control regimens were all considered eligible for inclusion:

Conventional rehabilitation therapy: As the predominant control type, it covered guideline-recommended routine stroke rehabilitation regimens including physical therapy, occupational therapy, neurodevelopmental therapy, gait training, and cognitive training, with matched session duration and training frequency relative to the VR exercise group.

Dose-matched active control training: Non-VR exercise with identical duration, frequency, and intensity to the intervention group, without any virtual reality-related technology components.

Sham/waitlist control: Standard care or sham stimulation (e.g., sham transcranial direct current stimulation, sham transcranial magnetic stimulation) combined with basic rehabilitation, with no active VR exercise components.

#### 2.1.4 Outcomes

Eligible outcomes were quantitative pooled endpoints, prioritizing RCT-derived data, with at least one core outcome required for inclusion.

##### 2.1.4.1 Motor function

Included indicators: Action Research Arm Test (ARAT), Fugl-Meyer Assessment (FMA, total/upper/lower extremity), Wolf Motor Function Test (WMFT), Functional Ambulation Category (FAC), Box and Blocks Test (BBT), and quantitative indicators related to manual dexterity.

##### 2.1.4.2 Balance and gait function

Included indicators: Gait velocity, comprehensive balance function, Berg Balance Scale (BBS), walking performance, step length, cadence, 10-Meter Walk Test (10-MWT), Timed Up and Go Test (TUG), and center of pressure (COP) parameters (velocity, path length, eyes open/closed).

##### 2.1.4.3 Cognitive function

Included indicators: Memory, executive function, visuospatial ability, Montreal Cognitive Assessment (MoCA), Mini-Mental State Examination (MMSE), Auditory/Visual Continuous Performance Tests (ACPT/VCPT), verbal fluency, and attention.

### 2.2 Study design

Included studies were peer-reviewed, published systematic reviews with meta-analyses focusing on VR exercise efficacy in post-stroke rehabilitation, which have completed quantitative pooling of the specified core outcomes. Priority was given to studies pooling only RCT data, and all eligible studies must comply with PRISMA 2020 guidelines with transparent reporting of study selection, data extraction, and quality assessment processes.

#### 2.2.1 Publication status and language restrictions

Only full-text, peer-reviewed journal articles were included to ensure methodological rigor, data completeness, and result transparency. Eligible studies were limited to English and Chinese publications, covering most high-quality studies in the field to minimize omission of core literature and avoid translation bias.

#### 2.2.2 Timeframe

Eligible systematic reviews and meta-analyses were formally published between January 1, 2010 and March 3, 2026, covering the entire application process of VR exercise in post-stroke rehabilitation (from initial exploration to the latest progress) to ensure the comprehensiveness, representativeness, and timeliness of the evidence.

### 2.3 Data sources and search strategy

Searches were conducted in 6 databases: PubMed, Embase, Web of Science Core Collection, Cochrane Library, CINAHL, and SPORTDiscus (January 1, 2010 to March 3, 2026; Figure 1). The search strategy was developed with an experienced researcher per PRESS guidelines, combining terms for stroke, VR exercise, systematic reviews/meta-analyses, and core outcomes. Reference lists of included studies were hand-searched. The full strategy is in Supplementary File S1.

**Figure 1:**
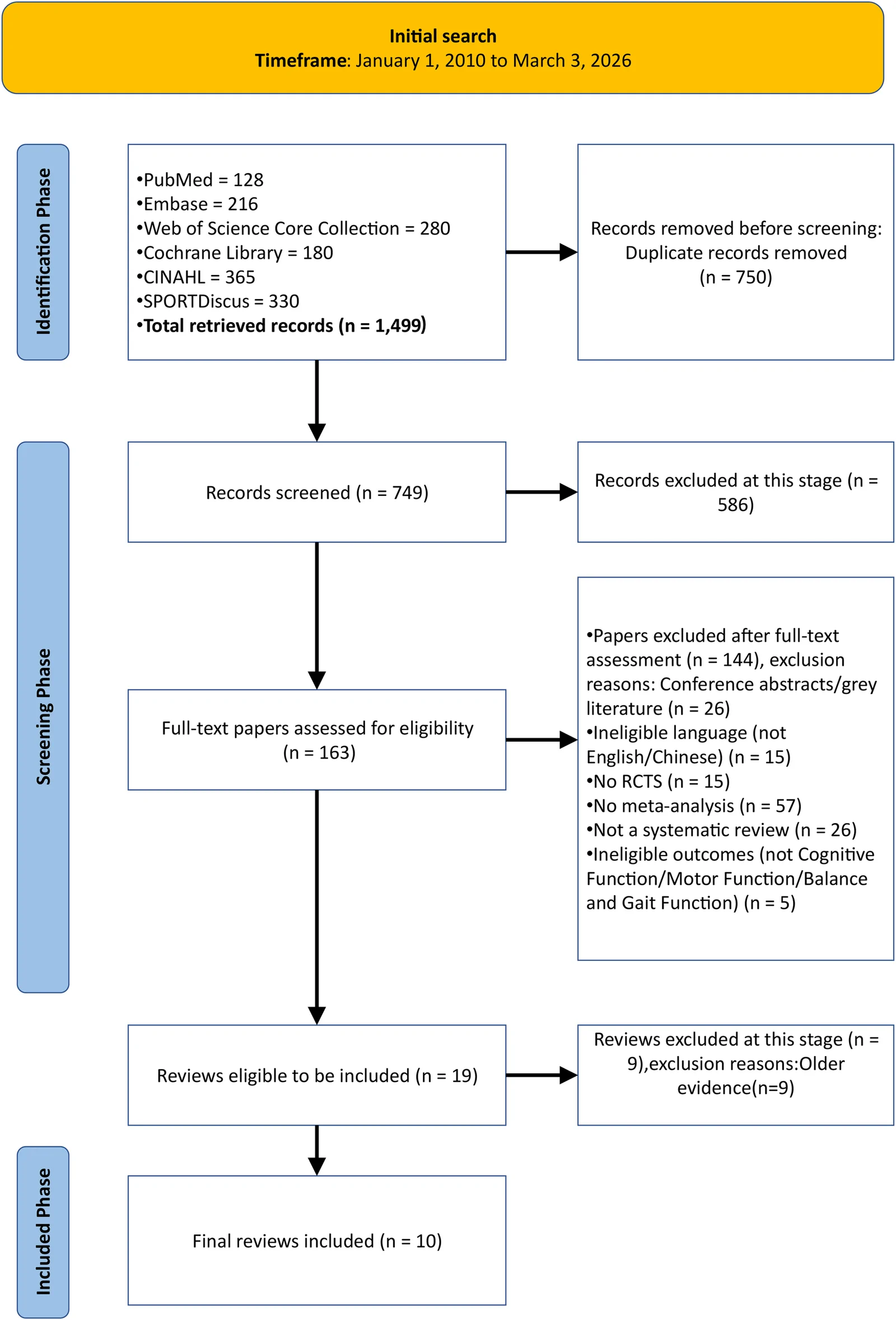
PRISMA flow diagram for study selection in the Umbrella review. Flow diagram of study identification, screening, eligibility, and inclusion process. A total of 1,499 records were retrieved from six databases; after duplicate removal and screening, 10 meta-analyses were included.

### 2.4 Study selection

Two reviewers (Y.G. and Y.C.) independently screened titles/abstracts, then full-texts, in duplicate. Discrepancies were resolved by discussion; unresolved cases were referred to a third reviewer (J.C.). The screening process included two sequential stages: title/abstract screening and full-text screening. In the first stage, reviewers independently screened all retrieved literature to preliminarily exclude studies clearly inconsistent with the research theme and design; potentially eligible studies were retained for full-text screening. In the second stage, reviewers independently read full texts, strictly verified against all inclusion/exclusion criteria, excluded ineligible studies, and recorded detailed, categorized exclusion reasons.

### 2.5 Data extraction

Two reviewers (Y.G. and Y.C.) performed duplicate data extraction using a pre-designed standardized form, which was pilot-tested on three eligible studies to optimize its rationality and completeness prior to formal use. Discrepancies were resolved through discussion; unresolved disagreements were referred to a third senior researcher (X.D.) for arbitration.

Data extraction was conducted at two hierarchical levels: basic characteristics of included meta-analyses, and individual data of primary RCTs. The specific extraction dimensions are as follows:

Basic characteristics of included meta-analyses: Extracted data included four core dimensions: (1) basic study characteristics (first author, publication year, country, included RCT sample size, stroke subtype distribution); (2) VR exercise intervention details (modality, equipment type, training parameters, combination strategies); (3) outcome data (core indicators, pooled effect sizes, 95% CIs, heterogeneity results, follow-up duration); (4) methodological characteristics (PRISMA 2020 compliance, bias assessment tools, meta-analysis statistical methods).

For each primary RCT, study identifiers, participant characteristics, intervention and comparator characteristics, outcome measures, assessment time points, and group-level numerical data (sample size, mean, and standard deviation) were extracted. These study-level characteristics were also used to identify duplicate RCTs across source meta-analyses. Risk-of-bias judgments for each RCT, extracted from the corresponding source reviews based on original authors’ standardized assessments, were subsequently used as inputs for the platform-generated GRADE-based evidence classification.

To improve the reliability and traceability of the *de novo* quantitative synthesis, only numerical data that could be verified from the eligible source reports or obtained from corresponding authors were used for effect-size calculation. When required group-level numerical data were incompletely reported, the corresponding authors were contacted where feasible. Studies for which the required numerical information remained unavailable after these procedures were excluded from the quantitative synthesis. These procedures were designed to minimize the risk of extraction error and selection related to differential data availability; however, residual bias arising from incomplete reporting could not be completely excluded. The complete RCT-level deduplication and data-availability screening process is summarized in Supplementary Table S4, while the individual primary RCTs excluded from the *de novo* quantitative synthesis and their specific exclusion reasons are reported in Supplementary Table S5.

### 2.6 Methodological quality assessment

The methodological quality for all included systematic reviews and meta-analyses was evaluated using AMSTAR 2 (A Measurement Tool to Assess Systematic Reviews 2), the internationally established gold-standard instrument (21). This tool includes 16 structured items, 7 of which are critical for determining overall quality grading. Two independent reviewers (Y.G. and Y.C.) received standardized training on AMSTAR-2 criteria, completed pre-assessment calibration with 10% of included studies, and then performed independent, parallel, double-blind duplicate assessments.

Each AMSTAR-2 item was rated “Yes”, “Partial Yes”, or “No” with clear documentation. Discrepancies were resolved through discussion; unresolved cases were referred to a third senior researcher. Quality grading was as follows: high (no critical flaws, ≤1 non-critical “Partial Yes”/“No”); moderate (no critical flaws, ≥2 non-critical “Partial Yes”/“No”); low (1 critical “No”, with/without non-critical flaws); critically low (≥2 critical “No”, with/without non-critical flaws). Assessment results supported evidence interpretation and sensitivity analysis, with detailed results in the Supplementary Materials.

### 2.7 Overlap of original studies between systematic reviews

Two independent reviewers (Y.G. and Y.C.) independently identified and quantified the overlap of original studies among included systematic reviews to avoid overlap bias and ensure result robustness. Overlap was determined by matching four key identifiers of original studies (title, first author, publication year, sample characteristics) to ensure accuracy.

The Corrected Covered Area (CCA) was used to quantify overlap, with the formula:

CCA=(N−r)/((r×c)−r)

Definitions: *N* = total number of the included primary studies in systematic reviews for the outcome (including double counting); *r* = number of unique primary studies; *c* = number of systematic reviews included for the outcome (22). The degree of overlap was categorized according to predefined criteria: mild (0%–5%), moderate (6%–10%), high (11%–15%), and very high (>15%). Discrepancies were resolved through discussion; unresolved cases were referred to a third senior researcher. All duplicated entries corresponding to identical RCTs were removed prior to quantitative synthesis, with only one unique record retained for each independent primary trial. This deduplication step eliminated correlated effect estimates induced by overlapping samples across multiple meta-analyses, and thus no additional statistical adjustment for inter-review correlation was required during subsequent pooled analysis. Detailed RCT-level deduplication accounting is provided in Supplementary Table S4.

### 2.8 Data synthesis

A combination of narrative and quantitative synthesis methods was used to comprehensively analyze the results of the included systematic reviews/meta-analyses. Study results were grouped by the three core outcome domains (motor function, balance and gait function, cognitive function) to ensure the clarity and comparability of results.

Narrative presentation: For the descriptive synthesis of source meta-analyses, the original direction of each reported effect size was retained. For the *de novo* quantitative synthesis, effect directions were interpreted according to the measurement characteristics of each outcome: positive eSMD values indicated a favorable effect for outcomes in which higher values represented better function, whereas negative eSMD values indicated a favorable effect for outcomes in which lower values represented better performance.

Quantitative synthesis: Primary RCTs may be represented across multiple systematic reviews addressing overlapping clinical questions, creating a risk of double counting when pooled estimates from overlapping reviews are combined directly. Methodological guidance for overviews recognizes that outcome data may be re-analyzed when appropriate to the objectives and analytical framework of the overview, while emphasizing the need to identify and address overlap among primary studies (23). Accordingly, the present review reconstructed the quantitative evidence at the primary-RCT level after identifying and removing duplicate trial records. This standardized approach was intended to reduce variation arising from differences in the statistical conventions and reporting practices of the source meta-analyses and to improve the comparability and traceability of the *de novo* estimates. All quantitative syntheses were performed using the deduplicated primary RCT dataset. Group-level means, standard deviations, and sample sizes were used to calculate equivalent standardized mean differences (eSMD) using a uniform computational procedure, followed by random-effects pooling with restricted maximum likelihood estimation of between-study variance. Statistical analyses were performed using the metaumbrella platform (https://metaumbrella.org/app) (24). This analytical pipeline removed the direct dependence introduced by duplicate representation of the same primary RCTs across multiple meta-analyses and thereby reduced the risk of duplicate weighting in the pooled estimates. Forest plots incorporating 95% confidence intervals were generated to visually present pooled eSMD values for each predefined primary outcome domain.

The resulting *de novo* pooled estimates, along with their associated heterogeneity, precision, and small-study-effect indicators (with *P* < 0.10 in Egger's regression defined as suggestive of potential bias), constituted the analytical inputs for the subsequent GRADE-based evidence classification.

### 2.9 GRADE-based evidence classification

The certainty of evidence for each pooled outcome was classified using the built-in GRADE-aligned algorithm of the metaumbrella platform, applied uniformly to all *de novo* synthesized datasets. The algorithm generates four ordinal evidence certainty categories in descending order: high, moderate, low, and very low. This classification follows core GRADE principles and was applied consistently across all outcomes; it should be distinguished from a full, manually adjudicated GRADE assessment. All ratings were derived from the characteristics of our re-synthesized evidence base rather than adopted directly from the source meta-analyses, ensuring consistent grading criteria across outcomes.

The procedure initially assigns a baseline rating of high certainty to all outcomes, and subsequently applies predefined downgrading criteria across four domains according to standardized, algorithmically defined thresholds, as specified in the platform's official tutorial. The specific downgrading rules are as follows:

Risk of bias (limitations): Downgrading is determined by the proportion of total participants enrolled in studies rated at low overall risk of bias. No downgrade is applied when ≥75% of participants come from studies at low risk of bias; one level is downgraded when the proportion falls between 50% and 75%; two levels are downgraded when the proportion is below 50%. Risk-of-bias judgments for individual RCTs were extracted directly from the included source systematic reviews, based on the original authors' standardized assessments. Given that this umbrella review reconstructs evidence from existing secondary syntheses rather than performing *de novo* methodological appraisal of primary trials, original study-level judgments were retained without retrospective recalibration across frameworks. Source reviews applied a range of risk-of-bias and quality instruments (predominantly the Cochrane risk-of-bias tool and the PEDro scale), which introduces inherent uncertainty into this domain and is acknowledged as a limitation.

Inconsistency: Inconsistency is quantified using the *I*^2^ statistic from the *de novo* random-effects meta-analysis. No downgrade is applied for *I*^2^ < 50%; one level is downgraded when *I*^2^ ≥ 50%.

Imprecision: Imprecision is evaluated based on the statistical power of the pooled sample to detect predefined standardized effect sizes. No downgrade is applied when power is ≥80% to detect a small effect size (Cohen's *d* = 0.20); one level is downgraded when power falls below 80% for a small effect; two levels are downgraded when power falls below 80% for a medium effect (Cohen's *d* = 0.50).

Publication bias: Publication bias and small-study effects are assessed via Egger's regression test for outcomes with ≥3 contributing studies. No downgrade is applied when Egger's *P* ≥ 0.10; one level is downgraded when Egger's *P* < 0.10. For outcomes with fewer than 3 studies, publication bias is classified as unevaluable and no downgrade is applied on this domain alone.

The built-in metaumbrella algorithm does not independently operationalize indirectness as a separate automatic downgrading domain. Potential clinical indirectness arising from differences in comparator conditions, outcome definitions, and assessment time points was therefore considered qualitatively when constructing and interpreting the *de novo* evidence base, rather than applied as an additional investigator-defined downgrade.

Two reviewers (Y.G. and Y.C.) independently verified all input data and final evidence classifications, with discrepancies resolved by discussion and third-reviewer (J.C.) adjudication. Outcome-specific evidence indicators and final GRADE-based classifications are presented in Supplementary Table S3.

## 3 Results

### 3.1 Study characteristics

A total of 10 meta-analyses focusing on the effects of virtual reality (VR) exercise on motor, balance and gait, and cognitive functional outcomes in stroke patients were included in this umbrella review (Supplementary Table S1). All included studies were published between 2020 and 2025, with a broad geographical distribution spanning China, Spain, the United Kingdom, South Korea, Finland, and Iran, with studies conducted in China accounting for the highest proportion (*n* = 5). All included meta-analyses were based on RCTs, with total participant sample size ranging from 493 to 3540, a median sample size of 12, 24, covering a total population of 14,352 patients. The total number of original RCTs included across all meta-analyses was 367, with individual meta-analyses including 11–87 primary studies.

The 10 included meta-analyses demonstrated overlapping coverage across the predefined outcome domains, with the specific distribution as follows: four focused exclusively on motor function outcomes; three restricted their analyses to balance and gait function outcomes; one addressed only the cognitive function domain; and two provided complete syntheses across all three core outcome domains—motor function, balance and gait, and cognitive function.

Regarding intervention comparisons, the included meta-analyses evaluated VR exercise under different clinical frameworks, including VR delivered as an adjunct to conventional rehabilitation and VR compared directly with conventional rehabilitation. Because these comparator frameworks address different clinical questions and were not consistently distinguished across the source meta-analyses, quantitative synthesis was performed according to functional outcomes rather than comparator categories. Accordingly, the pooled estimates reported in this review represent overall effects derived across heterogeneous comparator frameworks and should not be interpreted as a single homogeneous treatment effect.

A total of 367 primary RCT entries were identified across the 10 included source meta-analyses. This represented the pre-deduplication gross count, as the same primary trial could contribute to more than one source meta-analysis and outcome domain. The RCTs were subsequently organized according to the three prespecified functional domains. For motor function, 200 RCT entries were initially identified; 49 duplicate entries were removed, leaving 151 unique RCTs. A further 13 RCTs were excluded because the numerical information required for effect-size calculation was unavailable or incomplete, resulting in 138 RCTs available for the *de novo* motor-function analyses. For balance and gait function, 137 RCT entries were initially identified; 47 duplicate entries were removed, leaving 90 unique RCTs, of which 9 were subsequently excluded because the required numerical data were unavailable or incomplete, resulting in 81 RCTs for quantitative synthesis. For cognitive function, 37 RCT entries were identified, including 14 duplicate entries; after deduplication, 23 unique RCTs remained, and none required exclusion because of unavailable or incomplete numerical data. The complete accounting of RCT entries, duplicate records removed, unique trials retained after deduplication, and trials excluded because of unavailable or incomplete numerical data is provided in Supplementary Table S4. The individual RCTs excluded from the *de novo* quantitative synthesis and their specific reasons for exclusion are detailed in Supplementary Table S5. Because individual RCTs could contribute to more than one functional domain, the domain-specific counts are not mutually exclusive and therefore do not sum to the overall pre-deduplication total of 367 RCT entries.

The CCA index was adopted to quantify the degree of primary RCT overlap across included meta-analyses for each outcome domain. Higher CCA values indicate a greater proportion of identical primary trials repeatedly cited across multiple meta-analyses; direct pooling without prior deduplication would introduce bias from duplicate sample counting and distort the accurate estimation of true effect sizes. The CCA results and final counts of valid unique RCTs retained for quantitative synthesis per outcome domain are presented below:

Motor function domain: CCA = 6.49% (moderate overlap).
Balance and gait function domain: CCA = 13.06% (high overlap).
Cognitive function domain: CCA = 30.43% (very high overlap).

### 3.2 Narrative synthesis of included meta-analyses

This section describes the pooled effect estimates and methodological features reported in the 10 included meta-analyses, providing a baseline reference for the subsequent *de novo* quantitative synthesis based on deduplicated primary RCTs in this review.

Pooled effect estimates reported across the included studies indicated heterogeneous rehabilitative benefits of virtual reality exercise rehabilitation across the three predefined functional outcome domains. It should be clarified that no formal statistical comparison was conducted across different outcome domains in this review; the variations described below represent a descriptive summary of findings from original publications, reflecting only the inconsistency in statistical significance across individual outcome measures. Overall, source meta-analyses consistently reported positive effects of VR exercise on most motor, balance, and gait metrics, whereas pooled effects for multiple cognitive endpoints did not reach statistical significance. High between-study heterogeneity was a shared methodological characteristic across the included literature: 8 of the 10 source meta-analyses reported *I*^2^ values >50% for their primary outcomes, 6 core functional outcomes exhibited substantial to extreme heterogeneity (*I*^2^ ≥ 75%), and *I*^2^ values for some upper-extremity motor and memory metrics even exceeded 90%. This pervasive high heterogeneity constitutes a primary rationale for the subsequent subgroup analyses and the downgrading of GRADE certainty of evidence in this review.

### 3.3 Data synthesis and quality evaluation

#### 3.3.1 Standardized analytical pipeline

All quantitative syntheses were performed exclusively on the deduplicated dataset of non-overlapping primary RCTs. Using the sample size, mean value, and standard deviation (SD) of both the intervention and control groups extracted from each primary trial, equivalent standardized mean differences (eSMD) were uniformly calculated via the metaumbrella platform to enable standardized comparison of effect sizes across disparate assessment scales.

Convention for effect size direction: All pooled effect sizes in this study retained the original measurement direction of each functional scale, and no global manual sign reversal was implemented. This approach ensures full consistency between pooled results and the raw output of the metaumbrella platform, avoiding calculation errors and traceability bias introduced by manual conversion. Specifically, for positive-direction scales where higher values indicate better functional performance (e.g., FMA, BBS, gait velocity, MoCA), a positive eSMD indicates superior functional outcomes in the VR exercise group relative to the control group. For negative-direction scales where lower values indicate better functional performance (e.g., TUG completion time, COP sway velocity and path length), a negative eSMD indicates superior functional outcomes in the VR exercise group.

A random-effects model with restricted maximum likelihood (REML) estimation of between-study variance was applied for all overall and subgroup pooled analyses. All statistical computations were conducted using the web-based R package metaumbrella. Statistical significance was set at a two-tailed α level of 0.05, and a between-group difference was considered statistically significant if its 95% confidence interval (CI) did not cross the null value. Forest plots of overall pooled effects for all core outcomes are presented in Figure 2. Subgroup forest plots stratified by VR immersion type are displayed in Figures 3, 4, and those stratified by stroke stage are provided in Supplementary Figures S1–S3.

**Figure 2:**
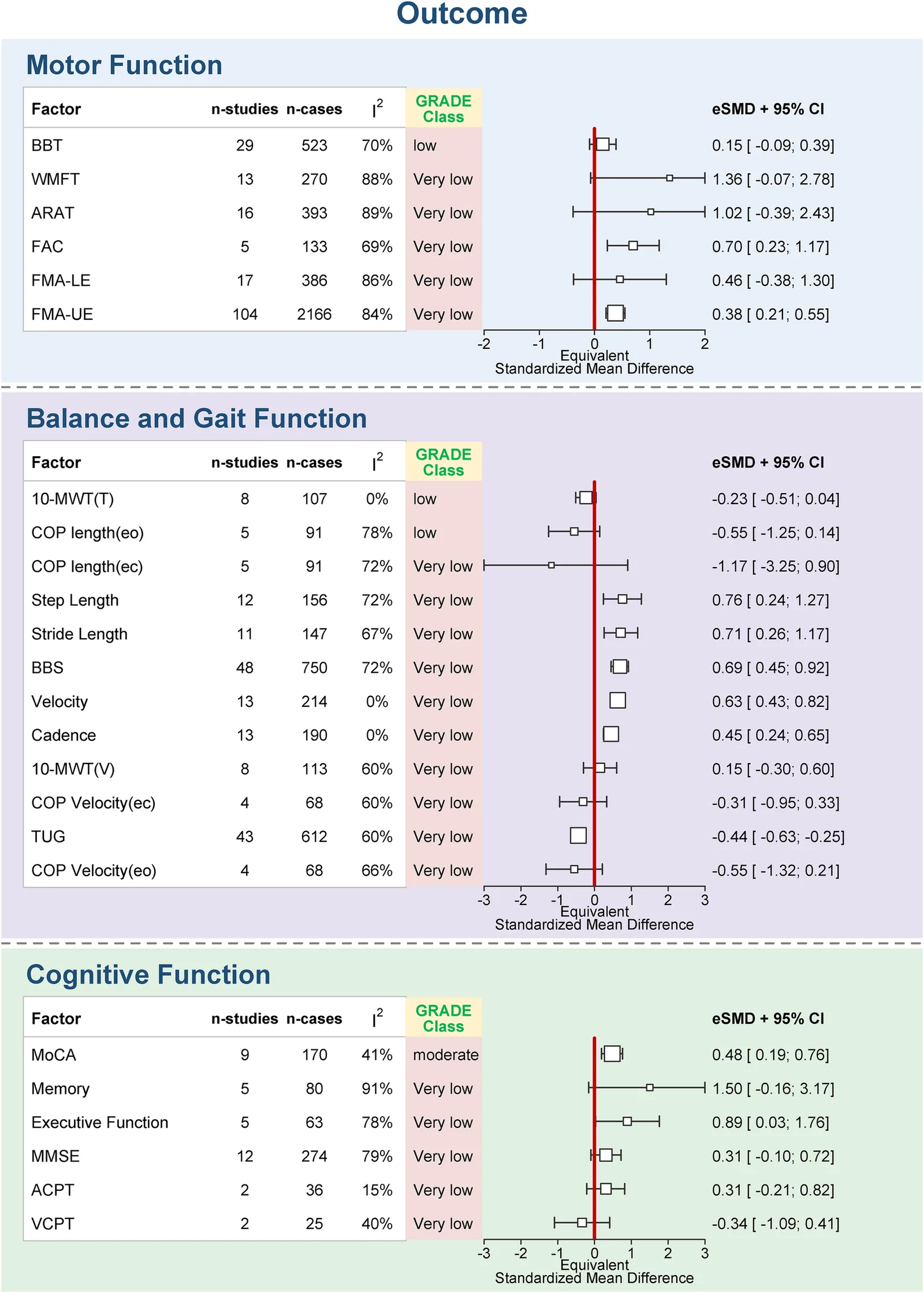
Forest plot of virtual reality exercise effects on motor, balance/gait, and cognitive function in stroke patients. Estimates are presented as eSMD with 95% CIs. Positive eSMD values indicate favorable effects for outcomes in which higher scores represent better function, whereas negative eSMD values indicate favorable effects for outcomes in which lower values represent better performance (e.g., TUG).

**Figure 3:**
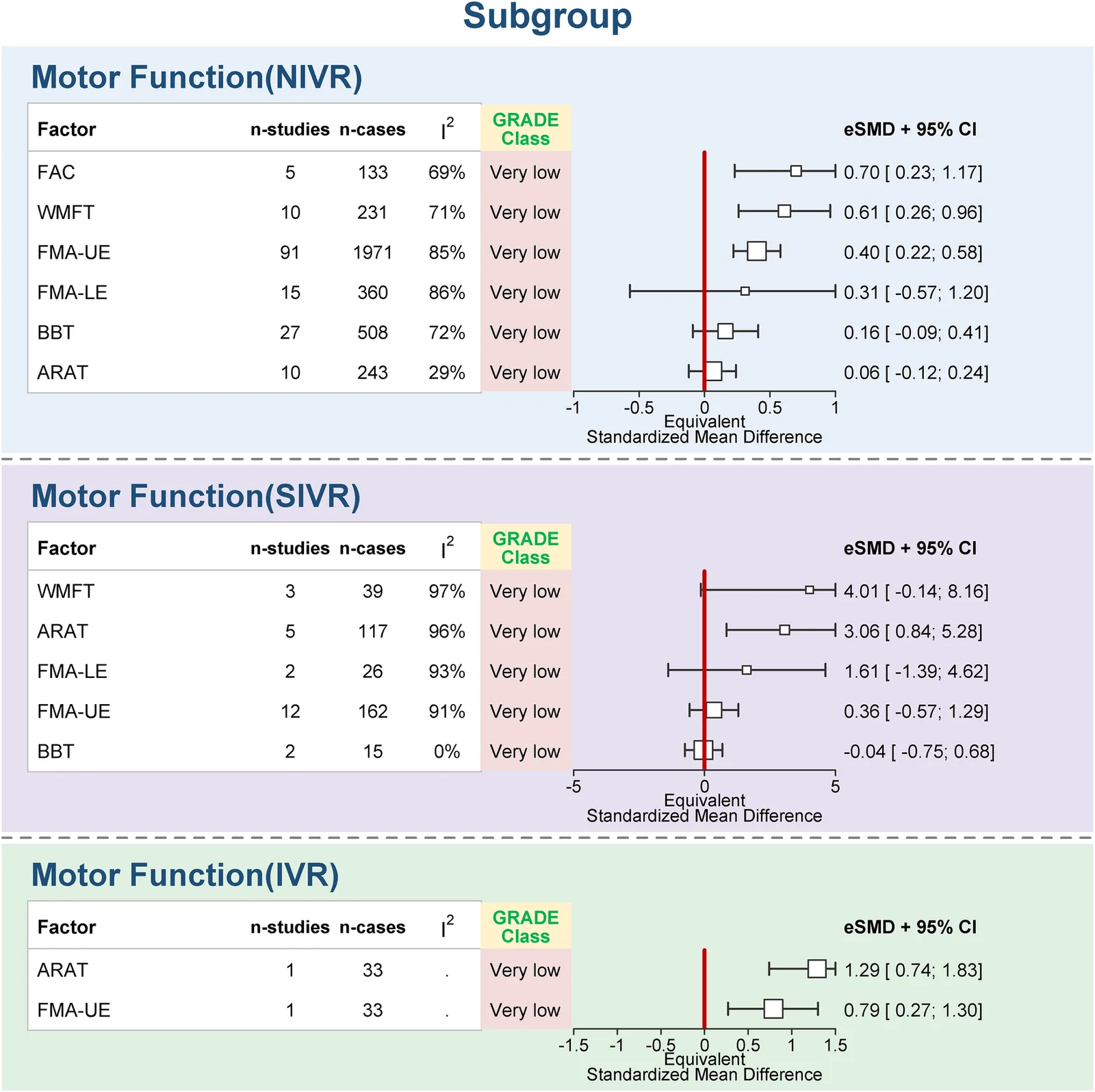
Forest plot of virtual reality exercise effects on motor function in stroke patients (subgroup of VR immersion type). Estimates are presented as eSMD with 95% CIs. Positive eSMD values indicate favorable effects for outcomes in which higher scores represent better function, whereas negative eSMD values indicate favorable effects for outcomes in which lower values represent better performance.

**Figure 4:**
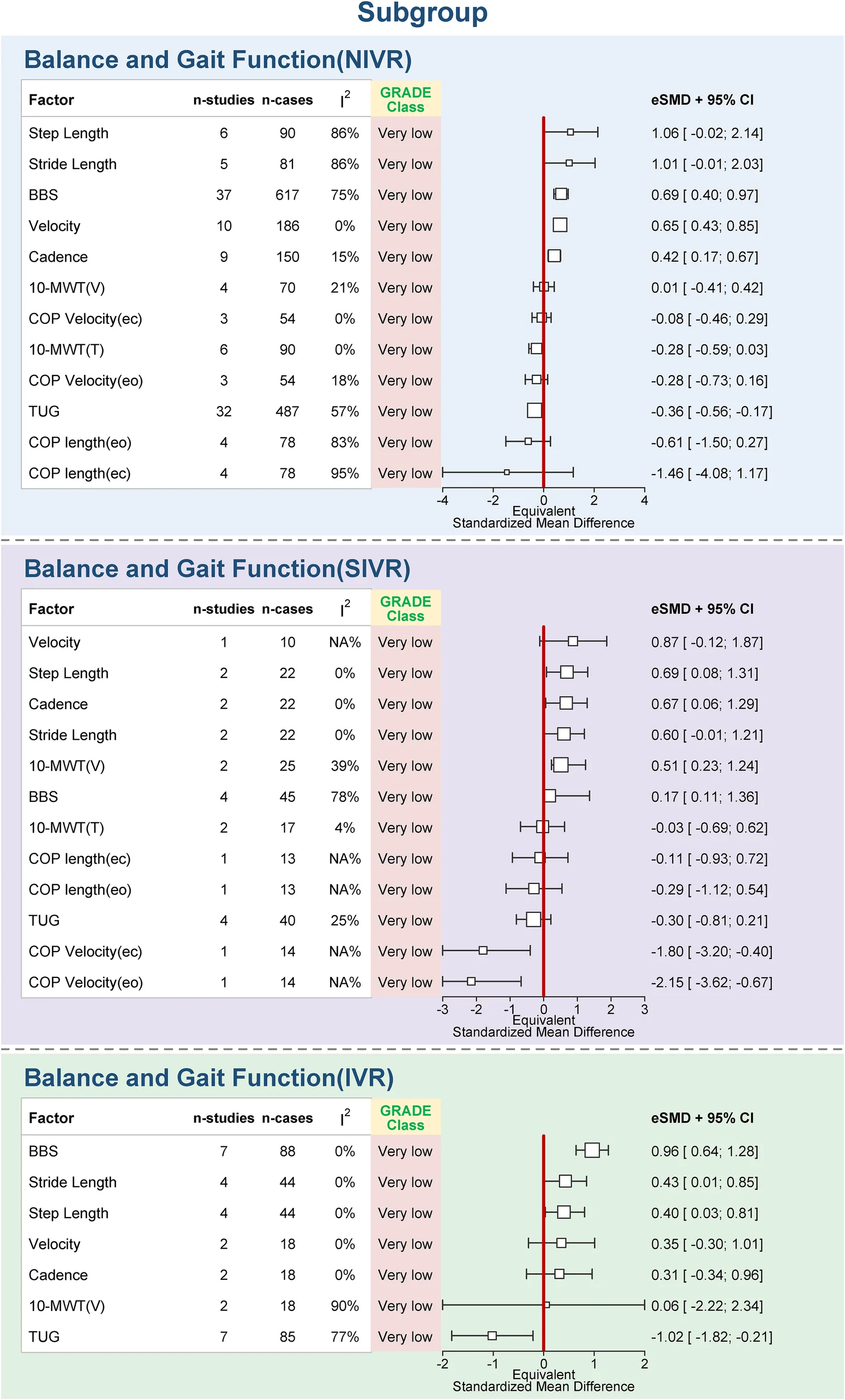
Forest plot of virtual reality exercise effects on balance and gait function in stroke patients (subgroup of VR immersion type). Estimates are presented as eSMD with 95% CIs. Positive eSMD values indicate favorable effects for outcomes in which higher scores represent better function, whereas negative eSMD values indicate favorable effects for outcomes in which lower values represent better performance.

A two-level evidence appraisal strategy was used. AMSTAR 2 was applied to assess the methodological quality of the included source systematic reviews, whereas the built-in GRADE criteria of the metaumbrella platform were used to generate GRADE-based evidence classifications for each *de novo* pooled outcome. The outcome-specific indicators and final evidence classifications are summarized in Supplementary Table S3.

#### 3.3.2 Motor function outcomes

The final deduplicated and data-availability-screened dataset for the motor function domain comprised 138 unique RCTs.

This subsection presents pooled effect estimates for the motor function domain, which comprises upper limb motor performance, lower limb motor function, and manual dexterity metrics. According to the GRADE-based classification, almost all motor function endpoints were graded as very low-certainty evidence; only the BBT was classified as low certainty.

FAC: A total of 5 RCTs (133 participants) were included. VR exercise significantly improved the functional ambulation category of stroke patients (eSMD = 0.70, 95% CI: 0.23–1.17), with moderate to high between-study heterogeneity (*I*^2^ = 69%).

FMA-UE: A total of 104 RCTs (2,166 participants) were included. VR exercise significantly improved upper limb motor function in stroke patients (eSMD = 0.38, 95% CI: 0.21–0.55), with extremely high between-study heterogeneity (*I*^2^ = 84%).

Four indicators showed a positive direction of effect without reaching statistical significance: FMA-LE (17 studies, 386 participants) with high between-study heterogeneity (*I*^2^ = 86%), ARAT (16 studies, 393 participants) with extremely high between-study heterogeneity (*I*^2^ = 89%), WMFT (13 studies, 270 participants) with extremely high between-study heterogeneity (*I*^2^ = 88%) and BBT (29 studies, 523 participants) with high between-study heterogeneity (*I*^2^ = 70%).

Numerical results from subgroup analyses indicated that pooled effect sizes for immersive and semi-immersive VR exercise were numerically larger than those for non-immersive protocols, and effect estimates for motor recovery were numerically greater in patients with chronic stroke relative to the subacute cohort. However, as no formal statistical comparisons between subgroups were performed, these observed differences remain purely descriptive and cannot be interpreted as evidence of definitive efficacy superiority across immersion modalities or stroke stages. Furthermore, substantial residual heterogeneity persisted within all stratified subgroups, suggesting that unmeasured clinical confounders may still contribute to variation in the pooled effect estimates.

#### 3.3.3 Balance and gait function outcomes

The final deduplicated and data-availability-screened dataset for the balance and gait function domain comprised 81 unique RCTs.

This subsection covers outcomes related to balance ability, gait parameters, and static postural control. According to the GRADE-based classification, the majority of balance and gait function endpoints were graded as very low-certainty evidence; only three indicators were classified as low certainty: 10-MWT (time), COP path length (eyes open), and COP path length (eyes closed).

Gait Velocity: A total of 13 RCTs (214 participants) were included. VR intervention significantly increased the gait velocity of stroke patients (eSMD = 0.63, 95% CI: 0.43–0.82), with no between-study heterogeneity (*I*^2^ = 0%).

BBS: A total of 48 RCTs (750 participants) were included. VR exercise significantly improved the balance performance of stroke patients as measured by the Berg Balance Scale (eSMD = 0.69, 95% CI: 0.45–0.92), with high between-study heterogeneity (*I*^2^ = 72%).

Step Length: A total of 12 RCTs (156 participants) were included. VR exercise significantly increased the step length of stroke patients during walking (eSMD = 0.76, 95% CI: 0.24–1.27), with high between-study heterogeneity (*I*^2^ = 72%).

Stride Length: A total of 11 RCTs (147 participants) were included. VR exercise significantly increased the stride length of stroke patients during walking (eSMD = 0.71, 95% CI: 0.26–1.17), with moderate between-study heterogeneity (*I*^2^ = 67%).

Cadence: A total of 13 RCTs (190 participants) were included. VR exercise significantly increased the cadence of stroke patients during walking (eSMD = 0.45, 95% CI: 0.24–0.65), with no between-study heterogeneity (*I*^2^ = 0%).

TUG: A total of 43 studies (612 participants) were included. VR exercise significantly shortened the completion time of the Timed Up and Go Test in stroke patients (eSMD = −0.44, 95% CI: −0.63 to −0.25), with moderate between-study heterogeneity (*I*^2^ = 60%).

Six indicators showed a positive effect direction without statistical significance. Between-study heterogeneity varied from none to extremely high (*I*^2^ = 0%–93%).

Stratified subgroup analyses demonstrated that immersive virtual reality yielded numerically larger pooled effect sizes for improvements in balance capacity and gait function, and that the magnitude of functional improvement was greater in the chronic stroke population relative to the subacute stroke cohort. However, as no formal statistical comparisons between subgroups were performed in this study, these observed differences represent purely descriptive findings and cannot be used to infer definitive superiority in therapeutic efficacy across immersion levels or stroke stages. No between-study heterogeneity was detected for gait velocity, step length, or cadence across all stratified subgroups.

Among stroke-stage subgroup analyses, only the BBS and gait velocity in the chronic stroke subgroup were graded as low GRADE certainty; all remaining balance and gait indicators carried very low-certainty evidence. Accordingly, only cautious, limited clinical implications can be derived, and no definitive, robust rehabilitation recommendations can be established.

#### 3.3.4 Cognitive function outcomes

The final deduplicated and data-availability-screened dataset for the cognitive function domain comprised 23 unique RCTs.

This section covers outcomes related to global cognitive function and specific cognitive subdomains. According to the GRADE-based classification, nearly all cognitive function endpoints were rated as very low-certainty evidence, with the MoCA representing the sole outcome graded as moderate certainty.

Executive Function: A total of 5 RCTs (63 participants) were included. VR exercise significantly improved executive function in stroke patients (eSMD = 0.89, 95% CI: 0.03–1.76), with high between-study heterogeneity (*I*^2^ = 78%).

MoCA: A total of 9 RCTs (170 participants) were included. VR exercise significantly improved the global cognitive function of stroke patients as measured by the Montreal Cognitive Assessment (eSMD = 0.48, 95% CI: 0.19–0.76), with moderate between-study heterogeneity (*I*^2^ = 41%).

Four indicators (Memory, MMSE, ACPT, VCPT) showed a positive effect direction without statistical significance. Between-study heterogeneity ranged from low to extremely high (*I*^2^ = 15%–91%).

#### 3.3.5 Publication bias and small-study effects

Most outcomes showed no clear evidence of small-study effects. Signals suggestive of small-study effects were observed for WMFT, ARAT, BBS, TUG, memory, executive function, and MMSE based on Egger's regression. These findings do not establish publication bias but indicate additional uncertainty surrounding the corresponding pooled estimates. For outcomes with fewer than three contributing studies, including ACPT and VCPT, Egger's regression could not be performed; therefore, the possibility of small-study effects could not be formally evaluated.

### 3.4 AMSTAR 2 quality assessment

The results showed that the overall quality was predominantly very low, with only a few studies rated as low quality; no studies were graded as high or moderate quality (Supplementary Table S2). Detailed quality classification is as follows:

Low quality (*n* = 2): Studies by Lu et al. (25) and Zhang et al. (26). These studies met most core AMSTAR 2 criteria, including clear PICO-based research questions, pre-specified protocols, comprehensive literature searches, duplicate independent screening and data extraction, detailed reporting of included studies, appropriate bias risk assessment, and adequate discussion of heterogeneity and publication bias. Their main limitations were the lack of a complete list of excluded studies with detailed reasons and unreported funding sources of included original studies.

Very low quality (*n* = 8, 80% of total): Studies by Villarroel et al. (27), Tian et al. (28), Krohn et al. (29), Soleimani et al. (30), Zhang et al. (31), Zhang et al. (32), Zhang et al. (33), and Lin et al. (34). Critical methodological flaws included unprespecified protocols, incomplete literature searches, lack of excluded study lists, unreported funding sources of original studies, and insufficient investigation of publication bias, leading to serious deficiencies in methodological integrity.

The most common methodological flaws were: (1) incomplete literature search strategies in over 80% of studies, leading to potential literature omission; (2) lack of complete excluded study lists with reasons in all studies, introducing potential selection bias; (3) unreported funding sources of original studies and inadequate disclosure of conflicts of interest in some studies, affecting result objectivity; (4) unprespecified protocols in 20% of studies, risking outcome reporting bias; (5) insufficient assessment of bias risk impact and inadequate discussion of high heterogeneity, reducing result reliability.

## 4 Discussion

This study systematically integrated 10 published meta-analyses and reconstructed a dataset based on deduplicated primary RCTs to perform quantitative analyses, providing a comprehensive evaluation of the effects of VR exercise on motor function, balance and gait function, and cognitive function in patients with stroke. Unlike conventional umbrella meta-analyses, the present study adopted a strategy of recalculating effect sizes after removing duplicate RCTs, to mitigate duplicate weighting bias caused by overlapping primary studies across different meta-analyses and thereby improve the independence and reliability of effect estimates.

### 4.1 Principal findings

Overall, favorable trends of VR exercise were observed across multiple motor function as well as balance and gait function indicators, and certain cognitive function endpoints also showed marginal improvements. Although most motor- and balance-related metrics demonstrated statistically significant improvements, the findings should be interpreted in light of the clinical meaningfulness reflected by specific assessment scales. Importantly, most outcomes were rated as very low-certainty evidence per the GRADE-based evidence classification, the majority of included meta-analyses were of critically low methodological quality per AMSTAR 2, and substantial statistical heterogeneity persisted across multiple endpoints. Collectively, these findings indicate that a high degree of uncertainty remains in the current evidence base.

As an umbrella review, this study not only re-summarized the therapeutic effects of VR exercise, but also comprehensively appraised the quality of the existing evidence base. The findings revealed that, despite a growing body of RCTs and meta-analyses accumulated in the field of VR-based stroke exercise, the overall quality of evidence remains limited. Marked discrepancies exist across studies in terms of VR device types, training protocols, intervention dosage, stroke recovery phase, assessment instruments, and statistical approaches, all of which may compromise the consistency of efficacy estimates. Accordingly, the present study concludes that current evidence generally supports VR exercise as a potentially valuable adjuvant intervention modality for stroke rehabilitation. Nevertheless, the true magnitude of its therapeutic efficacy and the optimal application strategies still require further verification through additional high-quality randomized controlled trials.

### 4.2 Motor function outcomes

The present study demonstrated that the effects of VR exercise on motor function in stroke patients were not uniformly consistent across outcome measures. Favorable effects were observed for FMA-UE and FAC, whereas FMA-LE, BBT, ARAT, and WMFT did not reach statistical significance. This variation suggests that the magnitude and consistency of the observed effects may depend on the specific dimension of motor function assessed.

Interpretation of the pooled estimates is further constrained by substantial between-study heterogeneity. In particular, heterogeneity was high for FMA-UE (*I*^2^ = 84%), FMA-LE (*I*^2^ = 86%), ARAT (*I*^2^ = 89%), and WMFT (*I*^2^ = 88%), indicating considerable variability in effect estimates across the contributing studies. Therefore, even where statistically significant improvements were observed, the corresponding pooled effects should be interpreted as average estimates across heterogeneous study populations rather than as consistent or universal effects of VR exercise for motor recovery.

Interpretation of the ARAT and WMFT findings warrants additional caution, as Egger's regression suggested potential small-study effects for both outcomes. These signals introduce additional uncertainty regarding the stability of the corresponding pooled estimates and should be considered alongside their substantial between-study heterogeneity.

This pattern is generally consistent with recent systematic reviews indicating that VR-based rehabilitation may facilitate motor recovery after stroke, while the magnitude and stability of effects vary according to assessment tools, intervention characteristics, and patient populations (14, 35–37). One possible explanation is that VR-based task-oriented exercise may facilitate activity-dependent neuroplasticity and repeated motor practice, which has been proposed as a mechanism for motor recovery following stroke (38, 39). Previous evidence has suggested that VR-based interventions may provide intensive, repetitive, and task-oriented practice with interactive feedback. Nevertheless, the specific neurobiological processes underlying these functional changes remain to be further elucidated. Further studies using standardized intervention protocols and clinically interpretable outcome measures are needed to clarify which dimensions of motor function are most responsive to VR exercise and under which clinical conditions.

### 4.3 Balance and gait function outcomes

The effects of VR exercise on balance and gait function also varied across assessment indicators. Statistically significant improvements were observed for gait velocity, BBS, step length, stride length, cadence, and TUG, whereas several other gait and postural-control measures did not reach statistical significance. The corresponding degree of between-study heterogeneity varied considerably across outcomes.

Gait velocity and cadence showed no observed statistical heterogeneity (both *I*^2^ = 0%), whereas substantial heterogeneity was observed for BBS and step length (both *I*^2^ = 72%), with moderate heterogeneity for stride length (*I*^2^ = 67%) and TUG (*I*^2^ = 60%). Several COP-related outcomes also showed marked heterogeneity, with *I*^2^ values reaching 93%. These differences indicate that the consistency of the observed effects was not uniform across balance and gait outcomes. Accordingly, pooled improvements in outcomes with substantial heterogeneity should be interpreted cautiously as average effects across the contributing studies rather than as broadly generalizable effects of VR exercise.

Although BBS and TUG showed statistically significant improvements, both outcomes were associated with signals suggestive of small-study effects in Egger's regression. These findings add uncertainty to the corresponding pooled estimates, particularly given the substantial heterogeneity observed for BBS (*I*^2^ = 72%) and moderate heterogeneity for TUG (*I*^2^ = 60%).

These findings are broadly consistent with previous systematic reviews indicating that VR-based gait and balance training may contribute to improvements in balance-related outcomes among stroke patients. However, previous evidence, including Cochrane reviews, has also highlighted uncertainty regarding its effects on certain mobility outcomes, such as walking speed. Therefore, although VR exercise appears promising as an adjunctive intervention for balance and gait rehabilitation, its effects should not be interpreted as uniformly superior across all gait and postural control measures (40, 41). The potential benefits of VR-based exercise may be partly explained by the provision of interactive feedback, task-oriented practice, and multisensory stimulation that may facilitate balance control and motor learning (42–45). Virtual gait tasks with progressive difficulty can gradually enhance patients' dynamic balance and sit-to-stand transfer function, directly translating into improvements in actual walking ability (46). Nevertheless, these mechanisms remain theoretical. Further high-quality studies are needed to determine the optimal VR exercise characteristics for improving specific balance and gait domains.

### 4.4 Cognitive function outcomes

Regarding cognitive outcomes, VR exercise was associated with statistically significant improvements in global cognitive function and executive function, whereas memory, MMSE, ACPT, and VCPT did not reach statistical significance. The magnitude and consistency of effects differed across cognitive outcomes.

Notably, executive function showed a relatively large pooled effect (eSMD = 0.89), but this estimate was accompanied by high between-study heterogeneity (*I*^2^ = 78%). Similarly, memory demonstrated very high heterogeneity (*I*^2^ = 91%) despite a non-significant pooled effect, whereas MoCA showed moderate heterogeneity (*I*^2^ = 41%). These findings indicate that the observed effects were not equally consistent across cognitive domains. In particular, the relatively large point estimate for executive function should not be interpreted as evidence of a stable or universal cognitive benefit of VR exercise, especially given the very low-certainty evidence for this outcome.

The memory outcome also warrants cautious interpretation. Although no statistically significant pooled effect was observed, the estimate was accompanied by evidence suggestive of small-study effects in Egger's regression. Given the very high heterogeneity (*I*^2^ = 91%), the current evidence is insufficient to determine whether the absence of a statistically significant pooled effect reflects a true lack of benefit or uncertainty arising from heterogeneous and potentially selective evidence.

For ACPT and VCPT, the number of contributing studies was insufficient for Egger's regression, and publication bias or small-study effects therefore could not be formally assessed. The corresponding findings should consequently be interpreted as uncertain rather than as evidence of an absence of publication bias.

These findings align with prior systematic reviews suggesting that technology-assisted rehabilitation, including VR-based cognitive and physical interventions, may contribute to improvements in post-stroke cognitive function. However, these reviews also emphasize that the available evidence remains preliminary due to limited sample sizes, variation in intervention designs, and insufficient standardization of cognitive training components. The present umbrella review further extends previous evidence by demonstrating differences in certainty across cognitive outcomes, with stronger evidence observed for global cognition than for specific cognitive subdomains (47). Several mechanisms have been proposed to explain the cognitive effects of VR rehabilitation, including increased cognitive engagement, dual-task demands, and visuospatial interaction during virtual tasks (48, 49). Virtual scene navigation and spatial judgment tasks can precisely target visuospatial neglect commonly seen after stroke (50–52). However, these mechanisms remain hypothetical. Future research should investigate whether specific VR exercise characteristics can selectively target different cognitive domains and determine the long-term clinical relevance of cognitive improvements after stroke.

### 4.5 Interpretation of methodological quality and evidence certainty

AMSTAR 2 assessment indicated that the methodological quality of the included systematic reviews was predominantly critically low. This finding reflects important limitations in review conduct and reporting and does not by itself determine the certainty of the *de novo* quantitative evidence.

The GRADE-based evidence classifications varied across outcomes. MoCA was classified as moderate-certainty evidence; BBT, 10-MWT (time), and COP path length were classified as low-certainty evidence; and most remaining outcomes were classified as very low-certainty evidence. These classifications were generated from the *de novo* pooled datasets using the predefined algorithm implemented in metaumbrella and were not directly inherited from the source meta-analyses. The principal factors reflected in the classifications included limitations in risk of bias, between-study inconsistency, imprecision, and potential small-study effects.

Statistical heterogeneity represents an important consideration in interpreting the pooled estimates. The extent of heterogeneity varied across functional domains and individual outcomes, with particularly high heterogeneity observed for several motor, balance and gait, and cognitive outcomes. Such variability may arise from differences in VR modality, training intensity and duration, stroke recovery stage, baseline functional status, comparator conditions, concurrent conventional rehabilitation, and outcome assessment instruments. These factors may contribute to inconsistency in treatment effects across studies and were therefore considered in the interpretation of evidence certainty. Overall, the pooled estimates should be understood as average effects across heterogeneous intervention contexts rather than as universal treatment effects applicable to all stroke populations.

Potential publication bias and small-study effects were considered when interpreting the credibility of the pooled estimates. Egger's regression showed no clear evidence of small-study effects for most outcomes; however, signals suggestive of small-study effects were observed for WMFT, ARAT, BBS, TUG, memory, executive function, and MMSE. These findings do not establish publication bias but indicate that the corresponding pooled estimates may be influenced by small-study-related factors and should therefore be interpreted cautiously. For outcomes with fewer than three contributing studies, Egger's regression could not be performed, and the possibility of small-study effects could not be formally evaluated. In particular, the absence of a formal test for ACPT and VCPT should not be interpreted as evidence that publication bias was absent.

In addition, this umbrella review evaluated the overlap of primary RCTs across included meta-analyses. The CCA index was calculated to quantify the degree of overlap within each outcome domain. CCA was used to describe the independence of the existing evidence base rather than as a criterion for excluding studies. Although overlap among primary studies existed for some outcomes, the present study minimized the potential impact of duplicate evidence by conducting *de novo* analyses based on deduplicated primary RCT data.

Overall, the current evidence suggests that VR exercise may provide potential benefits for motor function, balance and gait, and cognitive outcomes after stroke; however, the confidence in these findings is constrained by methodological limitations, substantial heterogeneity, possible publication bias, and generally limited evidence certainty. Because the evidence ratings were generated using an algorithmic GRADE-based procedure rather than a conventional manually adjudicated GRADE assessment, they should be interpreted within the analytical framework of the metaumbrella platform and not as a substitute for comprehensive domain-level GRADE judgments.

### 4.6 Clinical practice implications

Taken together, the current evidence suggests that VR exercise may represent a potentially useful component within comprehensive stroke rehabilitation strategies; however, the available evidence does not support replacing conventional rehabilitation with VR or prioritizing VR implementation for specific patient subgroups. Interpretation of the findings should consider the overall certainty of evidence, methodological limitations of the included reviews, substantial heterogeneity across outcomes, and differences in comparator conditions.

Importantly, statistically significant differences in pooled effect estimates should not be assumed to represent clinically meaningful benefits. For many functional outcomes assessed in this review, established minimal clinically important difference (MCID) thresholds are unavailable or inconsistently defined across assessment instruments, stroke recovery stages, and patient populations. Consequently, the clinical relevance of observed effect sizes cannot be determined from statistical significance alone.

The included evidence encompassed different clinical comparison frameworks, including VR delivered in addition to conventional rehabilitation and VR compared directly with conventional rehabilitation. These comparisons address distinct clinical questions, and the pooled estimates should therefore be interpreted as overall effects across heterogeneous comparator frameworks rather than as evidence of a single homogeneous treatment effect. Current findings are more appropriately interpreted as supporting the potential complementary role of VR within individualized rehabilitation strategies rather than its use as a substitute for established rehabilitation approaches.

From a practical perspective, the implementation of VR-based rehabilitation should be individualized according to patients' functional goals, tolerance, accessibility to equipment, available clinical resources, and rehabilitation context. Current evidence does not justify preferential implementation for specific patient subgroups. Future research should incorporate validated MCID thresholds, patient-reported outcomes, and longer-term functional follow-up to determine whether statistically observed changes translate into meaningful improvements in patients' functioning and quality of life.

### 4.7 Strengths and limitations

This study has several strengths. First, to the best of our knowledge, this is the first umbrella review to systematically evaluate the evidence base of VR exercise for post-stroke rehabilitation across three major functional domains—motor function, balance and gait function, and cognitive function—while performing *de novo* quantitative synthesis based on deduplicated primary RCT data.

Second, analyses were structured according to prespecified functional domains, with additional subgroup analyses based on VR immersion level and stroke recovery phase to explore potential variation in pooled effect estimates across intervention and patient characteristics.

Nevertheless, several limitations should be acknowledged. First, the methodological quality of the included systematic reviews was generally limited, and the quality and risk-of-bias judgments for the contributing primary RCTs were derived from source systematic reviews that used different assessment instruments. Although the original study-level judgments were retained for the *de novo* evidence synthesis, differences in assessment frameworks may limit direct comparability across reviews and introduce residual uncertainty into the risk-of-bias component of the evidence classification.

Second, substantial clinical and statistical heterogeneity existed across the included studies, with differences in VR technology, intervention protocols, training dosage, stroke recovery stage, baseline functional status, assessment instruments, and comparator frameworks. In particular, the evidence incorporated both VR as an adjunct to conventional rehabilitation and VR as a direct alternative to conventional rehabilitation. Because these comparison frameworks address different clinical questions and could not be consistently separated across the source meta-analyses, the pooled estimates may reflect a mixture of adjunctive and comparative effects.

Third, although this review performed *de novo* quantitative synthesis using deduplicated primary RCT data, the analytical framework limited the feasibility of certain advanced analyses, including meta-regression, prediction intervals, and some RCT-level sensitivity analyses. Consequently, residual sources of heterogeneity and uncertainty could not be fully explored.

Fourth, although trials with unavailable or incomplete numerical data were transparently documented and excluded according to predefined data-availability criteria, such exclusions may have introduced selection bias related to differential reporting completeness. This potential source of selection bias should therefore be considered when interpreting the *de novo* pooled estimates.

Fifth, the clinical interpretation of several pooled effect estimates was limited by the lack of consistently established MCID thresholds across assessment instruments and stroke populations. Consequently, statistical significance could not always be translated into clinically interpretable benefit.

Finally, several outcomes were supported by a limited number of RCTs, resulting in reduced statistical power and limited assessment of small-study effects. Egger's regression could not be performed for outcomes with fewer than three contributing studies, while selected outcomes showed signals suggestive of small-study effects. Therefore, publication bias cannot be excluded for several outcomes, and the corresponding pooled estimates should be interpreted cautiously.

### 4.8 Future research directions

Future research on VR rehabilitation in stroke should focus on the following priorities: (1) Conduct large-sample, multi-center, prospectively registered randomized controlled trials to standardize VR rehabilitation equipment, training dosage, and task paradigms; (2) Conduct long-term follow-up studies to clarify the persistence of VR rehabilitation benefits and their long-term impact on quality of life and independent living ability; (3) Conduct refined subgroup analyses by stroke phase, injury severity, and lesion location to construct precise rehabilitation programs; (4) Combine neuroimaging techniques to reveal the central neural mechanisms of VR-based synergistic physical-cognitive rehabilitation; (5) Conduct health economics studies to evaluate the cost-effectiveness of VR rehabilitation and promote its large-scale clinical application.

## 5 Conclusions

Findings from this umbrella review suggest that VR exercise may be associated with improvements in selected motor, balance and gait, and cognitive outcomes after stroke, but the magnitude and consistency of these effects remain uncertain. In the absence of established minimal clinically important difference (MCID) thresholds across assessment instruments and stroke populations, statistically significant findings cannot be interpreted as definitive clinically meaningful benefits, with interpretation further constrained by substantial heterogeneity, methodological limitations of the underlying evidence base, and generally low-certainty evidence. Current evidence supports VR exercise as a potential adjunctive rehabilitation approach, but does not justify its use as a replacement for conventional rehabilitation or preferential implementation in specific patient subgroups, and further high-quality randomized controlled trials incorporating standardized intervention protocols, validated clinically interpretable outcome thresholds, and long-term follow-up are therefore needed to clarify the clinical value and optimal implementation of VR-based rehabilitation.

## Data Availability

The original contributions presented in the study are included in the article/Supplementary Material, further inquiries can be directed to the corresponding author.
