# Supplementary material for: The impact of virtual reality exercise on motor function, balance and gait function, and cognitive function in stroke patients: an umbrella review of systematic reviews and meta-analyses
Source: Front Sports Act Living. 2026 Sep 17;8:1860232. doi: 10.3389/fspor.2026.1860232 (PMC13627310; doi:10.3389/fspor.2026.1860232)
Supplement: Supplementary file 1 [file Table1.docx]

Supplementary Material

**Supplementary File S1**

**Literature Search Strategy and Search Terms**

A targeted search strategy was designed for 6 electronic databases (PubMed, Embase, Web of Science Core Collection, Cochrane Library, CINAHL, SPORTDiscus), with a search period from January 1,2010 to March 3,2026. Search terms were combined around four core dimensions: "Population, Exposure, Outcomes, Study Design", and adjusted according to the characteristics of each database to ensure comprehensiveness and avoid missing relevant studies.

**I. Summary of Core Search Dimensions and Keywords**

Search terms were classified into four dimensions, which could be flexibly combined according to database retrieval rules (e.g., MeSH terms, subject headings, free text). Core keywords are as follows:

**1. Population**

Stroke, Cerebrovascular accident, Cerebral infarction, Cerebral hemorrhage, Ischemic stroke, Hemorrhagic stroke, Stroke patients, adult stroke survivors, Elderly stroke patients (human studies only)

**2. Exposure**

Virtual reality, VR, Virtual reality training, VR rehabilitation, Virtual reality exercise, VR-based intervention, Immersive virtual reality, non-immersive virtual reality, Virtual reality game, VR game

**3. Outcomes**

Motor function, Upper limb motor function, Lower limb motor function, Fugl-Meyer Assessment, FMA, Balance function, Berg Balance Scale, BBS, Gait function, Gait parameters, Step length, Stride length, walking speed, Timed Up and Go Test, TUG, Cognitive function, Global cognitive function, Executive function, Memory, Attention, Mini-Mental State Examination, MMSE, Montreal Cognitive Assessment, MoCA.

**4. Study Design**

Systematic review, Meta-analysis, Umbrella review, Systematic review with meta-analysis, Randomized Controlled Trial, RCT

**II. Specific Search Strategies and Keyword Combinations for Each Database**

Note: Keyword combinations for each database were connected using "AND" and "OR" logic. Keyword priority and expression were adjusted according to database characteristics. The complete search formulas for each database are listed below:

**1. PubMed**

(("Stroke"[MeSH Terms] OR "Cerebrovascular Accident"[MeSH Terms] OR "Cerebral Infarction"[Title/Abstract] OR "Cerebral Hemorrhage"[Title/Abstract] OR "Ischemic Stroke"[Title/Abstract] OR "Hemorrhagic Stroke"[Title/Abstract]) AND ("Virtual Reality"[MeSH Terms] OR "VR"[Title/Abstract] OR "Virtual Reality Training"[Title/Abstract] OR "VR Rehabilitation"[Title/Abstract] OR "Virtual Reality Exercise"[Title/Abstract]) AND ("Motor Function"[MeSH Terms] OR "Balance Function"[Title/Abstract] OR "Gait Function"[Title/Abstract] OR "Cognitive Function"[MeSH Terms] OR "Fugl-Meyer Assessment"[Title/Abstract] OR "Berg Balance Scale"[Title/Abstract] OR "Mini-Mental State Examination"[MeSH Terms]) AND ("Systematic Review"[MeSH Terms] OR "Meta-Analysis"[MeSH Terms] OR "Umbrella Review"[Title/Abstract] OR "Randomized Controlled Trial"[MeSH Terms]) AND ("2010/01/01"[Date - Publication] : "2026/03/03"[Date - Publication]) AND "Humans"[MeSH Terms])

**2. Embase**

("Stroke" OR "Cerebrovascular Accident" OR "Cerebral Infarction" OR "Cerebral Hemorrhage" OR "Ischemic Stroke" OR "Hemorrhagic Stroke" OR "Stroke Patient") AND ("Virtual Reality" OR "VR" OR "Virtual Reality Training" OR "VR Rehabilitation" OR "Virtual Reality Exercise" OR "VR-based Intervention") AND ("Motor Function" OR "Upper Limb Motor Function" OR "Lower Limb Motor Function" OR "Balance Function" OR "Gait Function" OR "Cognitive Function" OR "FMA" OR "BBS" OR "MMSE" OR "MoCA" OR "Activities of Daily Living") AND ("Systematic Review" OR "Meta-Analysis" OR "Umbrella Review" OR "Randomized Controlled Trial" OR "RCT") AND (2010/01/01 TO 2026/03/03) AND "Human"

**3. Web of Science Core Collection**

TS = ("Stroke" OR "Cerebrovascular Accident" OR "Cerebral Infarction" OR "Cerebral Hemorrhage" OR "Ischemic Stroke" OR "Hemorrhagic Stroke") AND TS = ("Virtual Reality" OR "VR" OR "Virtual Reality Training" OR "VR Rehabilitation" OR "Virtual Reality Exercise") AND TS = ("Motor Function" OR "Balance Function" OR "Gait Function" OR "Cognitive Function" OR "Fugl-Meyer Assessment" OR "Berg Balance Scale" OR "Cognitive Function" OR "Mini-Mental State Examination") AND TS = ("Systematic Review" OR "Meta-Analysis" OR "Umbrella Review" OR "Randomized Controlled Trial" OR "RCT") AND PY = (2010-2026) AND LA = English AND DT = (Review OR Article)

**4. Cochrane Library**

MeSH Terms: Stroke, Virtual Reality, Motor Function, Balance, Gait, Cognitive Function, Systematic Review, Meta-Analysis, Randomized Controlled Trial

Free Text: Cerebrovascular Accident, VR, Virtual Reality Training, VR Rehabilitation, FMA, BBS, MMSE, MoCA, Upper Limb Motor Function, Lower Limb Motor Function, Activities of Daily Living, Umbrella Review, RCT

Search Combination: (Stroke OR Cerebrovascular Accident OR Ischemic Stroke OR Hemorrhagic Stroke) AND (Virtual Reality OR VR OR Virtual Reality Training OR VR Rehabilitation) AND (Motor Function OR Balance Function OR Gait Function OR Cognitive Function OR FMA OR BBS OR MMSE OR MoCA) AND (Systematic Review OR Meta-Analysis OR Umbrella Review OR Randomized Controlled Trial OR RCT) AND Date: 2010-01-01 to 2026-03-03

**5. CINAHL**

(("Stroke"[Subject Headings] OR "Cerebrovascular Accidents"[Subject Headings] OR "Cerebral Infarction"[Text Word] OR "Cerebral Hemorrhage"[Text Word]) AND ("Virtual Reality"[Subject Headings] OR "VR"[Text Word] OR "Virtual Reality Training"[Text Word] OR "VR Rehabilitation"[Text Word]) AND ("Motor Function"[Subject Headings] OR "Balance"[Subject Headings] OR "Gait"[Subject Headings] OR "Cognitive Function"[Subject Headings] OR "Fugl-Meyer Assessment"[Text Word] OR "Berg Balance Scale"[Text Word] OR "Mini-Mental State Examination"[Subject Headings]) AND ("Systematic Reviews"[Subject Headings] OR "Meta-Analysis"[Subject Headings] OR "Umbrella Reviews"[Text Word] OR "Randomized Controlled Trials"[Subject Headings]) AND (PY = 2010-01-01 to 2026-03-03) AND "Humans"[Subject Headings])

**6. SPORTDiscus**

(("Stroke" OR "Cerebrovascular Accident" OR "Ischemic Stroke" OR "Hemorrhagic Stroke") AND ("Virtual Reality" OR "VR" OR "Virtual Reality Training" OR "VR Rehabilitation" OR "Virtual Reality Exercise") AND ("Motor Function" OR "Balance Function" OR "Gait Function" OR "Cognitive Function" OR "FMA" OR "BBS" OR "MMSE" OR "ADL") AND ("Systematic Review" OR "Meta-Analysis" OR "Umbrella Review" OR "Randomized Controlled Trial" OR "RCT") AND (2010-01-01 to 2026-03-03) AND "Human")

**III. Explanation of Search Strategy**

Keyword Selection: Based on the core theme of "the impact of virtual reality exercise on motor function, balance and gait, and cognitive function in stroke patients", standard terms in medical/rehabilitation science (e.g., MeSH terms, Subject Headings) were prioritized, supplemented with free text (e.g., "VR", "FMA", "BBS") and to reduce the risk of missing studies because of terminology differences.

Logical Combination: The core logic of "Population AND Exposure AND Outcomes AND Study Design" was adopted to ensure that retrieval results accurately meet the inclusion criteria of this umbrella review, focusing on systematic reviews, meta-analyses, and RCTs that explore VR intervention for stroke patients’ multi-dimensional functions.

Database Adaptation: Keyword priority and expression were adjusted according to the retrieval rules of each database (e.g., TS=topic terms for Web of Science, distinction between MeSH terms and free text for Cochrane Library, to improve retrieval efficiency and coverage.

Timeframe Setting: The search period was set from January 2010 to March 2026 to cover recent 16-year studies, ensuring the timeliness and comprehensiveness of literature.

Supplementary Table S1 Summary of Meta-Analyses Virtual Reality Exercise Effects on Motor, Balance/Gait, and Cognitive Function in Stroke Patients

| First Author | Year of Publication | Country | Population Description/Age | Stroke stage | Sample Size | Study Design | Exposure Measure Type (Absolute/Relative) | Specific Definition of Exposure Immersion  level | Intervention duration | Control Group Definition | Outcomes | Effect Size Type | Effect Size Value | 95% Confidence Interval (CI) | Subgroups (Rehabilitation Stage/VR Type/Intervention Duration, etc.) | Confounder Adjustment (Age/Stroke Course/BMI, etc.) | Number of Included Studies | AMSTAR 2 Quality Rating |
| --- | --- | --- | --- | --- | --- | --- | --- | --- | --- | --- | --- | --- | --- | --- | --- | --- | --- | --- |
| Rebeca Villarroel | 2025 | Spain | Stroke patients with upper limb motor dysfunction  aged 46.6~74.8 years | including acute subacute chronic | 1596 | RCT | Absolute (the direct effect of non-invasive VR intervention) | Non-invasive virtual reality (VR) rehabilitation intervention | Intervention frequency: 3 to 5 times per week, single session duration: 20 to 60 minutes, treatment course: 2 to 12 weeks | Conventional therapy (physical therapy, occupational therapy) | Upper limb motor function (FMA-UE);  Manual dexterity (BBT; MFT; WMFT; ARAT) | SMD | Upper limb motor function: SMD=5.24;  Manual dexterity: SMD=0.23; | Upper limb motor function: 2.76~7.73  Manual dexterity: 0.04~0.43 | Rehabilitation stage  Rehabilitation strategy  Intervention duration  VR interaction mode: Sensor  Game type | Yes | 40 | Very Low |
| Wenxin Lu | 2025 | UK | Stroke patients  aged ≥18 years | including subacute chronic | 768 | RCT | Absolute (the direct effect of VR intervention) | Virtual reality (VR) rehabilitation intervention: Immersive VR (IVR), Semi-Immersive VR (SIVR), Non-Immersive VR (NIVR) | Intervention frequency: 3 to 5 times per week, single session duration: 20 to 60 minutes, total treatment course: 3 to 12 weeks, total number of interventions: 10 to 40 times | Conventional rehabilitation therapy (physical therapy, gait training, balance training, etc.) | Balance function (BBS) mobility (TUG;10-MWT);  Gait parameters (step length, stride length) | MD | BBS: MD=3.29;  TUG: MD=-1.67;  10-MWT: MD=-0.91;  Step length: MD=3.59  Stride length: MD=5.63 | BBS:0.52~6.06  TUG: -2.89~-0.46  10-MWT:  -3.33~1.50  Step length:0.50~6.69  Stride length:  -0.73~11.99 | Stroke course  VR type  Intervention frequency | Yes | 24 | Low |
| Ming-Yu Tian | 2025 | South Korea | Stroke patients  aged ≥18 years | including acute subacute chronic | 1118 | RCT | Absolute (the direct effect of VR intervention) | Virtual reality (VR) rehabilitation intervention, including immersive and non-immersive VR | Intervention frequency: 3 to 5 times per week, single session duration: 20 to 60 minutes, total treatment course: 2 to 8 weeks | Conventional rehabilitation therapy, including physical therapy, balance training, traditional gait training | Static postural control (center of pressure, COP-related parameters: COP sway path length, COP velocity in eyes open/closed states);  Dynamic balance (BBS;TUG) | SMD  MD | Eyes open COP path length: SMD=-0.59;  Eyes closed COP path length: SMD=-0.54  Eyes open COP velocity: SMD=-0.38  Eyes closed COP velocity: SMD=-0.13  BBS: MD=3.29;  TUG: MD=-3.43 | Eyes open COP path length: -0.89~-0.28  Eyes closed COP path length: -0.87~-0.22  Eyes open COP velocity: -0.70~-0.05  Eyes closed COP velocity: -0.45~0.19  BBS: 2.76~3.83  TUG: -4.03~-2.82 | Intervention-related  Population-related:  Device-related: VR tool type  Study-related | Yes | 36 | Very Low |
| Maria Krohn | 2024 | Finland | Chronic stroke patients aged ≥18 years  mean age 59 years | chronic | 1136 | RCT | Absolute (the direct effect of VR combined with therapeutic exercise intervention) | Therapeutic exercise combined with virtual reality (VR) intervention: Low immersion, medium immersion, high immersion | The average intervention frequency is 3.5 times per week, the average duration of a single VR training session is 31.8 minutes (SD=10.0), and the average total course duration is 5.6 weeks. | Control measures without VR intervention, including 5 types: | Balance function (BBS; TUG);  Walking ability (10mWT; cadence) | SMD | Balance function: SMD=0.51;  Walking ability: SMD=0.31 | Balance function: 0.29~0.72  Walking ability: 0.09~0.53 | Intervention-related  Control-related  Device-related  Outcome-related | Yes | 35 | Very Low |
| Mohsen Soleimani | 2024 | Iran | Stroke patients  aged ≥18 years | including subacute chronic | 2142 | RCT | Absolute (the direct effect of VR intervention on upper limb function,) | Virtual reality (VR) rehabilitation intervention: Fully immersive, semi-immersive, non-immersive; | Intervention frequency: 3 to 5 times per week, single session duration: 20 to 60 minutes, treatment course: 2 days to 6 months | Conventional occupational therapy or physical therapy, | Upper limb motor function (FMA; ARAT; WMFT;  Dexterity (BBT) | SMD | Upper limb motor function:  FMA: SMD=0.63;  ARAT: SMD=1.56;  WMFT: SMD=0.93;  BBT: SMD=0.48 | FMA: 0.33~0.92  ARAT: 0.72~2.40  WMFT: 0.08~1.78  BBT: -0.05~1.02 | VR immersion level  Intervention duration  Stroke course: Subacute  Age  Intervention tools | Yes | 55 | Very Low |
| Nuo Zhang | 2024 | China | Stroke patients aged ≥18 years  mean age 43.6~73.4 years | including subacute chronic | 493 | RCT | Absolute (the direct effect of VR combined with NIBS intervention) | Virtual reality (VR) combined with non-invasive brain stimulation (NIBS) intervention | Frequency: 3 to 6 times per week, with a single VR training session lasting 20 to 60 minutes | Sham stimulation + VR  VR alone intervention;  Conventional rehabilitation therapy | Upper limb motor function (FMA-UE; WMFT); | SMD | FMA-UE: SMD=0.85;  WMFT: SMD=0.36 | FMA-UE: 0.40~1.31  WMFT: 0.08~0.64 (P=0.021) | Treatment duration  Stroke course  publication year  study type  age  total intervention duration | Yes | 11 | Low |
| Jiali Zhang | 2024 | China | Stroke patients aged ≥18 years  mean age 42.2~73.13 years | including subacute chronic | 1354 | RCT | Absolute (the direct effect of VR combined with conventional therapy) | Conventional therapy combined with virtual reality (VR) training: | Single training session duration: 30 to 120 minutes, training frequency: 2 to 5 times per week, total treatment course: 2 to 12 weeks | Conventional therapy alone (consistent with the type of conventional therapy in the intervention group) | Upper limb motor function (FMUE);  Manual dexterity (BBT) | SMD  MD | FMUE: SMD=0.32;  BBT: MD=2.79 | FMUE: 0.07~0.57  BBT: -0.63~6.20  FIM: -1.84~4.14 | Subgroup analysis based on stroke course | Yes | 27 | Very Low |
| Bohan Zhang | 2020 | China | Stroke patients aged ≥18 years  mean age of VR group 46.3~72.8 years, control group 47.5~76.4 years | Including acute subacute chronic | 3540 | RCT | Absolute (focusing on the direct effect of VR intervention) | Virtual reality (VR) rehabilitation intervention | Single duration: 15–100 minutes, frequency: 2–7 times per week, course of treatment: 2–12 weeks | Conventional rehabilitation therapy, including physical therapy (PT), occupational therapy (OT), conventional rehabilitation training, treadmill gait training | Limb motor function:(FMA-UE, ARAT, WMFT, BBTFMA-LE, FAC);  Balance and gait:( BBS, TUG, 10MWT, Velocity, Cadence)  Cognitive function:  (MMSE, ACPT; VCPT) | MD | FMA-UE: MD=6.75;  ARAT: MD=7.18;  WMFT: MD=4.43;  BBT: MD=1.73;  FMA-LE: MD=3.01;  FAC: MD=0.47;  BBS: MD=3.51;  TUG: MD=-2.10;  10MWT: MD=-1.45  Velocity: MD=11.79  Cadence: MD=8.35;  MMSE: MD=0.81;  ACPT: MD=0.03;  VCPT: MD=-0.03; | FMA-UE: 5.58~7.93  ARAT:4.27~10.08  WMFT: 2.46~6.40  BBT: -2.18~5.64  FMA-LE:1.91~4.11  FAC:0.14~0.79  BBS:2.10~4.92  TUG: -3.52~-0.73  10MWT:  -6.89~3.98  Velocity:8.48~15.11  Cadence:4.54~12.16  MMSE: -0.41~2.03  ACPT: -0.12~0.17  VCPT: -0.09~0.02 | Subgroup analysis stratified by VR intervention duration | Yes | 87 | Very Low |
| Qi Zhang | 2021 | China | Stroke patients aged ≥18 years | including acute subacute chronic | 894 | RCT | Absolute (the direct effect of VR intervention) | Virtual reality (VR) rehabilitation intervention, including immersive, semi-immersive, and non-immersive systems; | The intervention duration is 3 to 10 weeks (mainly 4 weeks), with a frequency of 2 to 5 times per week. | Conventional rehabilitation therapy, including conventional rehabilitation training, occupational therapy, computer-assisted cognitive rehabilitation, etc. | Cognitive function: Global cognitive function (MoCA/MMSE/LOTCA), Executive Function, Memory, Verbal Fluency, Visuospatial Function, Attention; | SMD | Global cognitive function: SMD=0.32  Executive Function: SMD=0.88  Memory: SMD=1.44  Verbal Fluency: SMD=0.11;  Visuospatial Function: SMD=0.78;  Attention: SMD=-0.09 | Global cognitive function: -0.43~1.06;  Executive Function: 0.06~1.70;  Memory: 0.21~2.68;  Verbal Fluency:  -0.38~0.61;  Visuospatial Function: 0.23~1.33;  Attention:  -0.39~0.22; | Preset subgroup analysis dimensions:  Control group type:  Intervention duration:  Assessment tools: | Yes | 23 | Very Low |
| Chenli Lin | 2023 | China | Elderly stroke patients aged ≥40 years | including acute subacute chronic | 1311 | RCT | Absolute (the direct effect of VR game intervention on cognition, mobility, and emotion,) | Virtual reality (VR) game rehabilitation intervention, | Single session duration: 15 to 120 minutes, frequency: 1.5 to 10 times per week, total treatment course: 1 to 12 weeks | Conventional rehabilitation therapy, including physical therapy (PT), occupational therapy (OT), cognitive training, health education, | Cognitive function: Global cognitive function, MMSE MoCA, Attention, Visuospatial Executive Function;  Mobility:(FMA BBS) | SMD  MD | Global cognitive function: MD=-8.79  MMSE: SMD=0.60  MoCA: MD=1.97  Attention: MD=0.25  Visuospatial Executive Function: MD=0.52  FMA: SMD=0.47  BBS: SMD=0.78 | Global cognitive function: -16.59~-1.00  MMSE: 0.26~0.95  MoCA: 1.30~2.64  Attention:0.01~0.49  Visuospatial Executive Function: 0.10~0.95  FMA: 0.02~0.93  BBS: 0.42~1.15 | No preset subgroup analysis was specified, but group analysis was conducted by comparing "VR games vs traditional rehabilitation training" to indirectly explore the impact of intervention type on outcomes | Yes | 29 | Very Low |

Note: This table summarizes the core information of 10 meta-analyses investigating the association between virtual reality (VR) intervention and multi-dimensional functional outcomes in stroke patients. ① for the type of exposure measurement, "Absolute" refers to directly quantified VR intervention effects (e.g., changes in scale scores, test results) with clear assessment indicators, focusing on the direct impact of VR on target functions. ② Regarding effect size types, Standardized Mean Difference (SMD) is used for outcomes with heterogeneous measurement tools across studies (to facilitate cross-study comparison), while Mean Difference (MD) is applied for outcomes with unified standardized scales/tests (to directly reflect absolute differences between groups). ③ A 95% CI that does not cross the null value indicates a statistically significant pooled effect at the two-sided α=0.05 level. ④ AMSTAR 2 quality ratings are categorized into four levels (High/Moderate/Low/Very Low) based on methodological completeness; higher ratings indicate greater reliability of study results. ⑤ Subgroup analyses cover dimensions such as rehabilitation stage, VR immersion level, intervention duration, and stroke course, while confounder adjustments include demographic characteristics (age, gender), disease-related indicators (stroke course, lesion type), and intervention-related parameters (training frequency, duration), ensuring the robustness of the association between VR intervention and outcomes.

Supplementary Table S2: Methodological Quality Assessment of Included Systematic Reviews/Meta-Analyses (Based on AMSTAR 2)

| First Author | Year of Publication | Country | Q1: Were the research question and inclusion criteria based on the PICO framework? | Q2: Were the systematic review methods pre-specified prior to implementation? Were inconsistencies with the study protocol explained? | Q3: Did the authors specify the types of studies included? | Q4: Did the authors adopt a comprehensive search strategy? | Q5: Was duplicate study selection conducted by two independent reviewers? | Q6: Was duplicate data extraction conducted by two independent reviewers? | Q7: Did the authors provide a list of excluded studies with corresponding reasons? | Q8: Did the authors describe the included studies in detail? | Q9: Did the authors assess the risk of bias of each included study using appropriate tools? | Q10: Did the authors report the funding sources of each included study? | Q11: For meta-analyses, did the authors use appropriate statistical methods to pool study results? | Q12: For meta-analyses, did the authors assess the potential impact of the risk of bias of included studies on meta-analytic results or other evidence synthesis outcomes? | Q13: Did the authors consider the risk of bias of included studies when interpreting or discussing results? | Q14: Did the authors reasonably explain and discuss any heterogeneity in study results? | Q15: For quantitative synthesis, did the authors fully investigate publication bias (small-study bias) and discuss its potential impact on results? | Q16: Did the authors report all potential sources of conflicts of interest, including any funding received for conducting the systematic review? | Overall Quality Rating (High/Moderate/Low/Very Low) |
| --- | --- | --- | --- | --- | --- | --- | --- | --- | --- | --- | --- | --- | --- | --- | --- | --- | --- | --- | --- |
| Rebeca Villarroel | 2025 | Spain | Yes | No | Yes | Partially Yes | Yes | Yes | Partially Yes | Yes | Yes | No | Yes | Yes | Yes | Yes | Yes | Yes | Very Low |
| Wenxin Lu | 2025 | UK | Yes | Yes | Yes | Yes | Yes | Yes | Partially Yes | Yes | Yes | No | Yes | Yes | Yes | Yes | Yes | Yes | Low |
| Ming-Yu Tian | 2025 | South Korea | Yes | Yes | Yes | Partially Yes | Yes | Yes | Partially Yes | Yes | Yes | No | Yes | Yes | Yes | Yes | Yes | Yes | Very Low |
| Maria Krohn | 2024 | Finland | Yes | Yes | Yes | Partially Yes | Yes | Yes | Partially Yes | Yes | Yes | No | Yes | Yes | Yes | Yes | Yes | Yes | Very Low |
| Mohsen Soleimani | 2024 | Iran | Yes | Yes | Yes | Partially Yes | Yes | Yes | Partially Yes | Yes | Yes | No | Yes | Yes | Yes | Yes | Yes | Yes | Very Low |
| Nuo Zhang | 2024 | China | Yes | Yes | Yes | Yes | Yes | Yes | Partially Yes | Yes | Yes | No | Yes | Yes | Yes | Yes | Yes | Yes | Low |
| Jiali Zhang | 2024 | China | Yes | Yes | Yes | Partially Yes | Yes | Yes | Partially Yes | Yes | Yes | No | Yes | Yes | Yes | Yes | Yes | Yes | Very Low |
| Bohan Zhang | 2020 | China | Yes | Yes | Yes | Partially Yes | Yes | Yes | Partially Yes | Yes | Yes | Yes | Yes | Yes | Yes | Yes | No | Yes | Very Low |
| Qi Zhang | 2021 | China | Yes | Yes | Yes | Partially Yes | Yes | Yes | Partially Yes | Yes | Yes | Yes | Yes | Yes | Yes | Yes | Partially Yes | Yes | Very Low |
| Chenli Lin | 2023 | China | Yes | No | Yes | Partially Yes | Yes | Yes | Partially Yes | Yes | Yes | Yes | Yes | Yes | Yes | Yes | Partially Yes | Yes | Very Low |

Note:

This table evaluates the methodological quality of 10 included studies based on the 16 core criteria of AMSTAR 2 (A Measurement Tool to Assess Systematic Reviews 2). ① Q1-Q16 represent the core assessment dimensions of AMSTAR 2: "Yes" indicates compliance with the criterion, "No" indicates non-compliance, and "Partially Yes" indicates partial compliance (considered non-compliant). ② Overall quality ratings are classified into four levels: "High" means full compliance with all 16 criteria, with the highest result credibility; "Moderate" means 3-4 non-compliant criteria without critical methodological flaws; "Low" means 5-6 non-compliant criteria; "Very Low" means ≥7 non-compliant criteria, including critical flaws such as failure to report reasons for excluded studies and failure to assess publication bias. ③ The Notes column indicates special circumstances of some studies, which can assist in interpreting the quality rating results

Supplementary Table S3 Summary of de novo pooled estimates and GRADE-based evidence classifications

| Outcome | Factor | eSMD | 95%CI | I^2^ | Egger’s p-value | Power indicator | Risk-of-bias indicator | GRADE-based classification |
| --- | --- | --- | --- | --- | --- | --- | --- | --- |
| Motor | BBT | 0.149 | [-0.092, 0.39] | 70.126 | 0.101 | Low es = 89.2;  Med. es = 100;  Large es = 100 | 50.29 | low |
| Motor | WMFT | 1.358 | [-0.068, 2.783] | 87.594 | 0.000443 | Low es = 63.7;  Med. es = 100;  Large es = 100 | 28.92 | Very low |
| Motor | ARAT | 1.02 | [-0.393, 2.434] | 88.65 | 0.0593 | Low es = 79.6;  Med. es = 100;  Large es = 100 | 20.15 | Very low |
| Motor | FAC | 0.699 | [0.226, 1.172] | 68.851 | 0.659 | Low es = 36.2;  Med. es = 98;  Large es = 100 | 0 | Very low |
| Motor | FMA-LE | 0.457 | [-0.382, 1.296] | 86.125 | 0.228 | Low es = 79.1;  Med. es = 100;  Large es = 100 | 0 | Very low |
| Motor | FMA-UE | 0.381 | [0.211, 0.551] | 83.91 | 0.351 | Low es = 100;  Med. es = 100;  Large es = 100 | 38.59 | Very low |
| Balance/Gait | 10-MWT(T) | -0.233 | [-0.506, 0.039] | 0 | 0.355 | Low es = 31;  Med. es = 95.5;  Large es = 100 | 58.8 | low |
| Balance/Gait | COP length(eo) | -0.553 | [-1.25, 0.143] | 77.776 | 0.769 | Low es = 26.8;  Med. es = 91.7;  Large es = 100 | 86.19 | low |
| Balance/Gait | COP length(ec) | -1.172 | [-3.246, 0.903] | 92.884 | 0.277 | Low es = 26.8;  Med. es = 91.7;  Large es = 100 | 86.19 | low |
| Balance/Gait | Step Length | 0.758 | [0.244, 1.273] | 72.445 | 0.174 | Low es = 42;  Med. es = 99.3;  Large es = 100 | 40.19 | Very low |
| Balance/Gait | Stride Length | 0.714 | [0.259, 1.169] | 67.311 | 0.375 | Low es = 40;  Med. es = 98.9;  Large es = 100 | 36.52 | Very low |
| Balance/Gait | BBS | 0.687 | [0.453, 0.921] | 72.16 | 0.00714 | Low es = 97;  Med. es = 100;  Large es = 100 | 57.13 | Very low |
| Balance/Gait | Velocity | 0.629 | [0.434, 0.825] | 0 | 0.785 | Low es = 54.5;  Med. es = 99.9;  Large es = 100 | 25 | Very low |
| Balance/Gait | Cadence | 0.445 | [0.238, 0.651] | 0 | 0.632 | Low es = 49.3;  Med. es = 99.8;  Large es = 100 | 24.01 | Very low |
| Balance/Gait | 10-MWT(V) | 0.15 | [-0.3, 0.599] | 59.538 | 0.96 | Low es = 31.8;  Med. es = 96.1;  Large es = 100 | 29.15 | Very low |
| Balance/Gait | COP Velocity(ec) | -0.312 | [-0.954, 0.331] | 59.553 | 0.405 | Low es = 19.9;  Med. es = 79.3;  Large es = 99.3 | 100 | Very low |
| Balance/Gait | TUG | -0.438 | [-0.626, -0.251] | 60.412 | 0.00162 | Low es = 93.6;  Med. es = 100;  Large es = 100 | 67.11 | Very low |
| Balance/Gait | COP Velocity(eo) | -0.553 | [-1.322, 0.215] | 66.313 | 0.51 | Low es = 19.9;  Med. es = 79.3;  Large es = 99.3 | 100 | Very low |
| Cognition | MoCA | 0.478 | [0.193, 0.762] | 40.814 | 0.613 | Low es = 47;  Med. es = 99.7;  Large es = 100 | 100 | Moderate |
| Cognition | Memory | 1.502 | [-0.161, 3.165] | 91.353 | 0.0293 | Low es = 24.4;  Med. es = 88.5;  Large es = 99.9 | 100 | Very low |
| Cognition | Executive Function | 0.891 | [0.026, 1.756] | 78.447 | 0.0712 | Low es = 20.3;  Med. es = 80.4;  Large es = 99.5 | 100 | Very low |
| Cognition | MMSE | 0.31 | [-0.102, 0.722] | 79.36 | 0.0972 | Low es = 64.5;  Med. es = 100;  Large es = 100 | 100 | Very low |
| Cognition | ACPT | 0.308 | [-0.207, 0.824] | 14.604 | < 3 studies | Low es = 13.1;  Med. es = 54;  Large es = 91 | 100 | Very low |
| Cognition | VCPT | -0.34 | [-1.095, 0.415] | 39.963 | < 3 studies | Low es = 10.4;  Med. es = 39.5;  Large es = 77.4 | 100 | Very low |

Note：GRADE-based classifications were generated using the built-in GRADE criteria of the metaumbrella platform. I² represents statistical heterogeneity; Egger’s p-value reflects the small-study-effect assessment; outcomes with fewer than three contributing studies were not formally assessable using Egger’s regression. eSMD, equivalent standardized mean difference; CI, confidence interval; I², heterogeneity statistic; GRADE, Grading of Recommendations Assessment, Development and Evaluation.

Supplementary Table S4 Primary RCT deduplication and data-availability screening for de novo quantitative synthesis

| Outcome | Initial RCT entries | Unique RCTs after deduplication | Duplicate entries removed | Excluded for unavailable/incomplete numerical data | Final RCTs analyzed |
| --- | --- | --- | --- | --- | --- |
| Motor | 200 | 151 | 49 | 13 | 138 |
| Balance/Gait | 137 | 90 | 47 | 9 | 81 |
| Cognition | 37 | 23 | 14 | 0 | 23 |

Supplementary Table S5 Primary RCTs excluded from de novo quantitative synthesis and reasons for exclusion

| First author | Year | Outcome | Source meta-analysis | Exclusion reason |
| --- | --- | --- | --- | --- |
| LeeMM | 2018 | Motor | Rebeca Villarroel | incomplete numerical data |
| Adomavičienė | 2019 | Motor | Mohsen Soleimani | incomplete numerical data |
| Ballester | 2017 | Motor | Mohsen Soleimani | incomplete numerical data |
| Doost | 2020 | Motor | Mohsen Soleimani | incomplete numerical data |
| Faria | 2018 | Motor | Mohsen Soleimani | incomplete numerical data |
| Fluet | 2014 | Motor | Mohsen Soleimani | incomplete numerical data |
| Hung | 2021 | Motor | Mohsen Soleimani | incomplete numerical data |
| Subramanian | 2013 | Motor | Mohsen Soleimani | incomplete numerical data |
| Huang | 2022 | Motor | Mohsen Soleimani | incomplete numerical data |
| Fei Huang | 2019 | Motor | Bohan Zhang | incomplete numerical data |
| Wenfeng Li | 2018 | Motor | Bohan Zhang | incomplete numerical data |
| Yana Li | 2018 | Motor | Bohan Zhang | incomplete numerical data |
| Lili Miao | 2016 | Motor | Bohan Zhang | incomplete numerical data |
| de Rooij | 2021 | Balance and Gait | Wenxin Lu | incomplete numerical data |
| Druzbicki | 2010 | Balance and Gait | Maria Krohn | incomplete numerical data |
| Fritz | 2013 | Balance and Gait | Maria Krohn | incomplete numerical data |
| Hung | 2017 | Balance and Gait | Maria Krohn | incomplete numerical data |
| Pedreira da Fonseca | 2017 | Balance and Gait | Maria Krohn | incomplete numerical data |
| Shin | 2016 | Balance and Gait | Maria Krohn | incomplete numerical data |
| Yu | 2016 | Balance and Gait | Maria Krohn | incomplete numerical data |
| Jonsdottir | 2021 | Balance and Gait | Maria Krohn | incomplete numerical data |
| Subramaniam | 2014 | Balance and Gait | Chenli Lin | incomplete numerical data |

Figure S1. Forest Plot of Virtual Reality Exercise Effects on Motor Function in Stroke Patients (subgroup of Stroke Staging‌). Estimates are presented as eSMD with 95% CIs. Positive eSMD values indicate favorable effects of VR exercise for outcomes in which higher scores represent better function, whereas negative eSMD values indicate favorable effects for outcomes in which lower values represent better performance.

Figure S2. Forest Plot of Virtual Reality Exercise Effects on Balance and Gait Function in Stroke Patients (subgroup of Stroke Staging‌). Estimates are presented as eSMD with 95% CIs. Positive eSMD values indicate favorable effects of VR exercise for outcomes in which higher scores represent better function, whereas negative eSMD values indicate favorable effects for outcomes in which lower values represent better performance.

Figure S3. Forest Plot of Virtual Reality Exercise Effects on Cognitive Function in Stroke Patients (subgroup of Stroke Staging‌). Estimates are presented as eSMD with 95% CIs. Positive eSMD values indicate favorable effects of VR exercise for outcomes in which higher scores represent better function, whereas negative eSMD values indicate favorable effects for outcomes in which lower values represent better performance.
